# Attaching and effacing (A/E) lesion formation by enteropathogenic *E*. *coli* on human intestinal mucosa is dependent on non-LEE effectors

**DOI:** 10.1371/journal.ppat.1006706

**Published:** 2017-10-30

**Authors:** Massiel Cepeda-Molero, Cedric N. Berger, Alistair D. S. Walsham, Samuel J. Ellis, Simon Wemyss-Holden, Stephanie Schüller, Gad Frankel, Luis Ángel Fernández

**Affiliations:** 1 Department of Microbial Biotechnology. Centro Nacional de Biotecnología, CNB-CSIC. Darwin 3, Campus UAM, Cantoblanco, Madrid, Spain; 2 MRC Centre for Molecular Bacteriology and Infection, Department of Life Sciences, Imperial College London, London, United Kingdom; 3 Norwich Medical School, University of East Anglia, Norwich, United Kingdom; 4 Gut Health and Food Safety Programme, Quadram Institute Bioscience, Norwich, United Kingdom; 5 Department of Surgery, Norfolk and Norwich University Hospital NHS Foundation Trust, Norwich, United Kingdom; McMaster University, CANADA

## Abstract

Enteropathogenic *E*. *coli* (EPEC) is a human pathogen that causes acute and chronic pediatric diarrhea. The hallmark of EPEC infection is the formation of attaching and effacing (A/E) lesions in the intestinal epithelium. Formation of A/E lesions is mediated by genes located on the pathogenicity island locus of enterocyte effacement (LEE), which encode the adhesin intimin, a type III secretion system (T3SS) and six effectors, including the essential translocated intimin receptor (Tir). Seventeen additional effectors are encoded by genes located outside the LEE, in insertion elements and prophages. Here, using a stepwise approach, we generated an EPEC mutant lacking the entire effector genes (EPEC0) and intermediate mutants. We show that EPEC0 contains a functional T3SS. An EPEC mutant expressing intimin but lacking all the LEE effectors but Tir (EPEC1) was able to trigger robust actin polymerization in HeLa cells and mucin-producing intestinal LS174T cells. However, EPEC1 was unable to form A/E lesions on human intestinal *in vitro* organ cultures (IVOC). Screening the intermediate mutants for genes involved in A/E lesion formation on IVOC revealed that strains lacking non-LEE effector/s have a marginal ability to form A/E lesions. Furthermore, we found that Efa1/LifA proteins are important for A/E lesion formation efficiency in EPEC strains lacking multiple effectors. Taken together, these results demonstrate the intricate relationships between T3SS effectors and the essential role non-LEE effectors play in A/E lesion formation on mucosal surfaces.

## Author summary

Enteropathogenic *E*. *coli* (EPEC) causes diarrhea and generates the attaching and effacing (A/E) lesion in human gut epithelium. A/E lesion formation requires the locus of enterocyte effacement (LEE) in the bacterial genome, which encodes a protein injection system delivering the translocated intimin receptor (Tir), which binds to intimin on the bacterial surface. Intimin-Tir interaction is sufficient for bacterial attachment to epithelial cells *in vitro* but additional effectors may be needed for A/E lesion formation in the human gut. By generating deletion mutants lacking combinations or the whole repertoire of protein effectors encoded by EPEC, we show that intimin-Tir interaction is not sufficient and reveal an additive role of non-LEE effectors for A/E lesion formation in human intestinal tissue.

## Introduction

The gastrointestinal epithelium is an important defense barrier against infections [1]. Enteric pathogens have acquired virulence traits that enable them to colonize and break this barrier, by adhering to the epithelium, delivering toxins and invading intestinal epithelial cells. To this end, several important human and animal pathogens employ type III secretion systems (T3SS) to inject virulence factors into infected eukaryotic cells, where they take control of cell signaling [2].

Enteropathogenic *E*. *coli* (EPEC) and enterohemorrahgic *E*. *coli* (EHEC) are important human pathogens that colonize the gut mucosa through attaching and effacing (A/E) lesions [3], characterized by intimate bacterial attachment to the apical plasma membrane, localized accumulation of F-actin and effacement of the brush border microvilli [4]. The ability to induce A/E lesions requires the pathogenicity island the locus of enterocyte effacement (LEE) [5, 6]. The LEE encodes gene regulators, the adhesin intimin, chaperones, a filamentous T3SS composed of the translocators proteins (EspA, EspB and EspD), and six effectors (Tir, EspF, Map, EspG, EspH, and EspZ) [7]. In HeLa cells, clustering of intimin with its receptor Tir [8] triggers robust actin polymerization leading to formation of pedestal-like structures [4, 9]. On mucosal surfaces, intimin–Tir interaction is necessary for A/E lesion formation, but it is not currently known if this binding is sufficient [10].

Most LEE effectors, except EspZ, are strong inducers of cytotoxicity, cytoskeleton reorganization, and electrolyte imbalance leading to diarrhea [11, 12]. Map functions as a Cdc42 GEF (Guanine nucleotide exchange factor), leading to filopodia formation on HeLa cells within minutes after infection [13, 14]; EspH inhibits the activity of endogenous DH-PH RhoGEFs causing disassembly of focal adhesions (FAs) and cell detachment [15, 16] and EspG interferes with recycling endosomes [17, 18]. EspZ, which like Tir integrates into the plasma membrane, regulates effector translocation, thus protecting infected cells form cytotoxicity [19].

The prototype EPEC strain E2348/69 also contains 17 effector genes located in integrative elements (IEs) and prophages (PPs). These effectors are frequently found in gene clusters, with some effectors having duplicated gene copies and/or paralogs in different clusters [20]. A large proportion of the non-LEE effectors (e.g. NleB, C, D, E, F and H) inhibits host inflammation ([e.g. nuclear factor kappa B (NF-κB); mitogen-activated protein kinase (MAPK) and the non-canonical inflammasome] [12, 21, 22] and apoptosis (e.g. NleB, D and H) [23]. In particular, NleC is a zinc metalloprotease that degrades the p65 subunit of NF-κB [24].

Deng et al. reported two additional non-LEE effectors, NleJ and LifA/Efa1 [25]. While the function of NleJ is not known, LifA/Efa1, also called lymphostatin [26], has a putative glycosyltransferase activity and an important role in intestinal colonization of cattle by EHEC serogroup O5, O111, and O26 strains [27–29], as *efa1* mutations dramatically reduced the number of mucosal associated bacteria and fecal shedding. The reason for this apparent attenuation is not known.

EPEC is a human restricted pathogen; for this reason human intestinal *in vitro* organ cultures (IVOC) have been used to study early interactions of EPEC with mucosal surfaces [30–33]. Following IVOC infection EPEC triggers A/E lesions that are indistinguishable from those observed in intestinal biopsies of patients with EPEC diarrhea. Using this model it has been shown that while intimin and Tir are essential for colonization, Tir tyrosine phosphorylation is dispensable for A/E lesion formation [10]. However, this infection model has not yet been used to investigate if intimin-Tir interaction is sufficient for A/E lesion formation. The aim of our study is to determine whehter intimin-Tir interaction is sufficient for A/E lesion formation in human IVOC identify further effector(s) required for their formation. To this end, we generated an effector-less mutant of E2348/69 strain and a library of intermediate deletion mutants lacking effectors and preserving the correct assembly and function of the T3SS injectisome. This unveiled that EPEC mutants expressing only Tir were unable to produce A/E lesions on IVOC, while able to produce typical actin pedestals on epithelial cells *in vitro*. In addition, we found that an EPEC mutant lacking all the non-LEE effector genes shows a marginal ability to form A/E lesions on human intestinal IVOC.

## Results

### Generation of the effector mutant EPEC strains

We employed a marker-less deletion/replacement strategy to generate a library of EPEC effector mutants, which allow multiple deletions and/or integrations while leaving neither an antibiotic gene cassette nor short heterologous DNA sequences ("scars") in the chromosome [34]. The mutant alleles were designed to delete the coding sequences of effector genes from the start to the stop codon, or in the case of gene clusters and operons, from the start codon of the first open reading frame (ORF) to the stop codon of the last ORF, maintaining upstream and downstream sequences containing endogenous regulatory elements (e.g. promoters, transcriptional terminators) intact (S1 Fig). We first tested this marker-less deletion strategy by generating an EPEC mutant in *escN*, encoding the ATPase of the T3SS, whose deletion abrogated secretion of EspA, EspB and EspD in DMEM, but not of EspC autotransporter (S2 Fig). Next, we generated a set of suicide vectors (pGE and pGETS derivatives) for the deletion of all the known effectors in E2348/69 (Table A of S1 Text) [20, 25].

**Fig 1:**
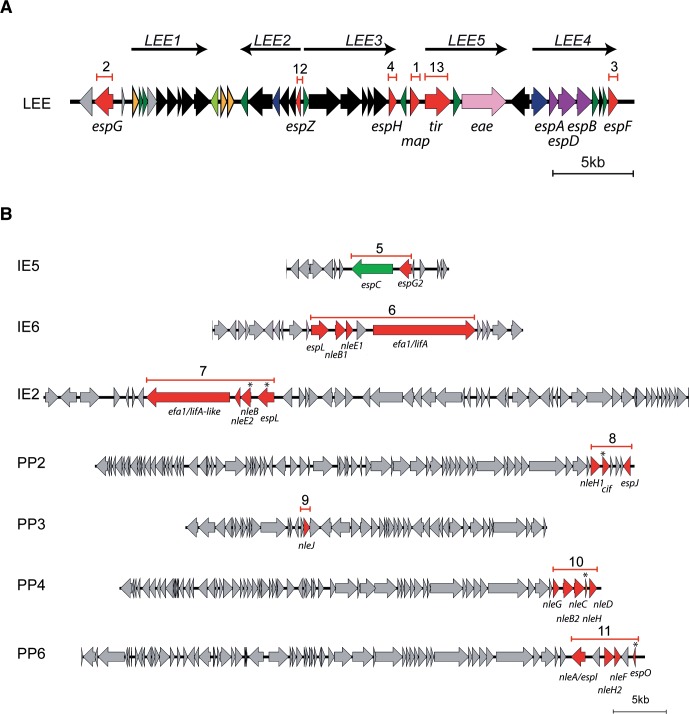
Deletion of effector genes in EPEC E2348/69. **A**. Representation of the LEE pathogenicity island. Effector genes are labeled in red. The order of deletion is numbered: deletion 1, 2, 3, 4 are for *map*, *espG*, *espF* and *espH* respectively. The *espZ* and *tir* genes were deleted after the non-LEE effectors, with order 12 and 13 respectively. Scale of 5 kb is indicated at the bottom. **B**. Effector genes located outside the LEE are localized in integrative elements (IE) and prophages (PP). Effector genes are labeled in red. Pseudogenes are specified with asterisk. The red bars indicate the deletions carried out. The order of deletion is numbered. Scale of 5 kb is indicated at the bottom.

We sequentially deleted LEE effector genes *map*, *espG*, *espF* and *espH* (Fig 1A) to obtain the mutant strain called EPEC9 (Table 1). The LEE effectors *espZ* and *tir* were not deleted at this stage, as EspZ, by regulating effector translocation, protects cells from cytotoxicity [19] and Tir, by mediating intimate attachment, enhances protein translocation [35].

**Table 1:** EPEC strains used in this work.

Strain	Genotype and relevant properties	Reference
**E2348/69**	Wild type EPEC O127:H6	[20]
**EPECΔ*escN***	E2348/69 Δ*escN*	This work
**EPECΔ*map***	E2348/69 Δ*map*	This work
**EPEC11**	E2348/69 Δ*map* Δ*espG*	This work
**EPEC10**	E2348/69 Δ*map* Δ*espG* Δ*espF*	This work
**EPEC9**	E2348/69 Δ*map* Δ*espG* Δ*espF* Δ*espH*	This work
**EPEC8**	EPEC9 ΔIE5 (*espG2 espC*)	This work
**EPEC7**	EPEC8 ΔIE6 (*espL*, *nleB1*, *nleE1*, *efa1*/*lifA*)	This work
**EPEC6**	EPEC7 ΔIE2 (espL*, nleB*, *nleE2*, *efa1*/*lifA*-like)	This work
**EPEC5**	EPEC6 ΔPP2 (*nleH1*, cif*, *espJ*)	This work
**EPEC4**	EPEC5 ΔPP3 (*nleJ*)	This work
**EPEC3**	EPEC4 ΔPP4 (*nleG*, *nleB*, *nleC*, nleH*, *nleD*)	This work
**EPEC2**	EPEC3 ΔPP6 (*nleA*/*espI*, *nleH2*, *nleF*, espO*)	This work
**EPEC1**	EPEC2 Δ*espZ-2*	This work
**EPEC0**	EPEC1 Δ*tir*	This work
**EPEC2Δ*eae***	EPEC2 Δ*eae*	This work
**EPEC1Δ*eae***	EPEC1 Δ*eae*	This work
**EPEC2*map***	EPEC2 +*map*	This work
**EPEC1*map***	EPEC1 +*map*	This work
**EPEC0*map***	EPEC0 +*map*	This work
**EPEC2*nleC***	EPEC2 +*nleC*	This work
**EPEC1*nleC***	EPEC1 +*nleC*	This work
**EPEC0*nleC***	EPEC0 +*nleC*	This work
**EPEC2*LEE***	EPEC2 +*map*, +*espH*, +*espF*, +*espG*	This work
**EPEC7Δ*lifA*-like**	EPEC7 Δ*efa1/lifA*-like	This work
**EPEC7Δ*nleE2***	EPEC7 Δ*nleE2*	This work
**EPECΔ*lifA*-like**	E2348/69Δ*lifA-*like	This work
**EPECΔ*lifA***	E2348/69Δ*lifA*	This work
**EPECΔ*lifA*-likeΔ*lifA***	E2348/69Δ*lifA-*like Δ*lifA*	This work

Next, we deleted the genes encoding the non-LEE effectors (Fig 1B). The order of deletion followed was: IE5 (*espG2* and *espC*), IE6 (*espL*, *nleB1*, *nleE1*, *efa1*/*lifA*) and IE2 (espL*, nleB*, *nleE2*, *efa1*/*lifA*-like). Although EspC is not a T3SS effector, we deleted *espC* together with *espG2* in the IE5 because EspC has been reported to be internalized into the host cell in a T3SS-dependent manner [36, 37], can interact with translocon proteins [38], and is known to induce severe cytopathic effects and cell death on epithelial cells [39, 40]. The resulting effector mutant strains were called EPEC8, EPEC7 and EPEC6, respectively (Table 1).

We continued by sequential deletion of the effector genes in PPs: PP2 (*nleH1*, cif*, *espJ*), PP3 (*nleJ*), PP4 (*nleG*, *nleB*, *nleC*, nleH*, *nleD*) and PP6 (*nleA*/*espI*, *nleH2*, *nleF*, espO*) (Fig 1B), resulting in a strain we named EPEC2, which contains EspZ and Tir as the only effectors. We then proceed with the deletion of *espZ*. However, we found that deletion of the coding sequence of *espZ* (Δ*espZ*-1, S3A Fig), which is the first gene of the *LEE2* operon, reduced the secretion of the translocators (EspA, EspB and EspD) of the T3SS (S3B Fig). We speculated that abortive translation initiation induced by the RBS of *espZ* could potentially affect translation of downstream genes in the *LEE2* operon. Then, we generated a second mutant allele of *espZ* that included deletion of its RBS, called Δ*espZ*-2 (S3A Fig), which did not affect secretion of the translocators (S3B Fig). Using this mutant allele on EPEC2, we generated the EPEC1 strain that only carries *tir*. Lastly, we deleted *tir* in EPEC1 generating the effector-less strain EPEC0. The steps followed to delete T3 effectors in WT EPEC are summarized in Table B of S1 Text.

During generation of each mutant strain, we confirmed the expected deletion by PCR using specific primers (Table C). Confirmation of all deletions in EPEC0 is shown in S4 Fig. In addition, we performed whole-genome sequencing of the parental WT EPEC and EPEC1. Sequencing reads were assembled both using the reference genomes of EPEC E2349/69 and the *in silico* designed sequence of EPEC1, as well as fully assembled *de novo* from the sequencing reads. Genome comparison between WT EPEC and EPEC1 showed that the only differences between both strains were the designed deletions (Table D in S1 Text).

### The T3SS injectisome is functional in the EPEC effector mutants

**Fig 2:**
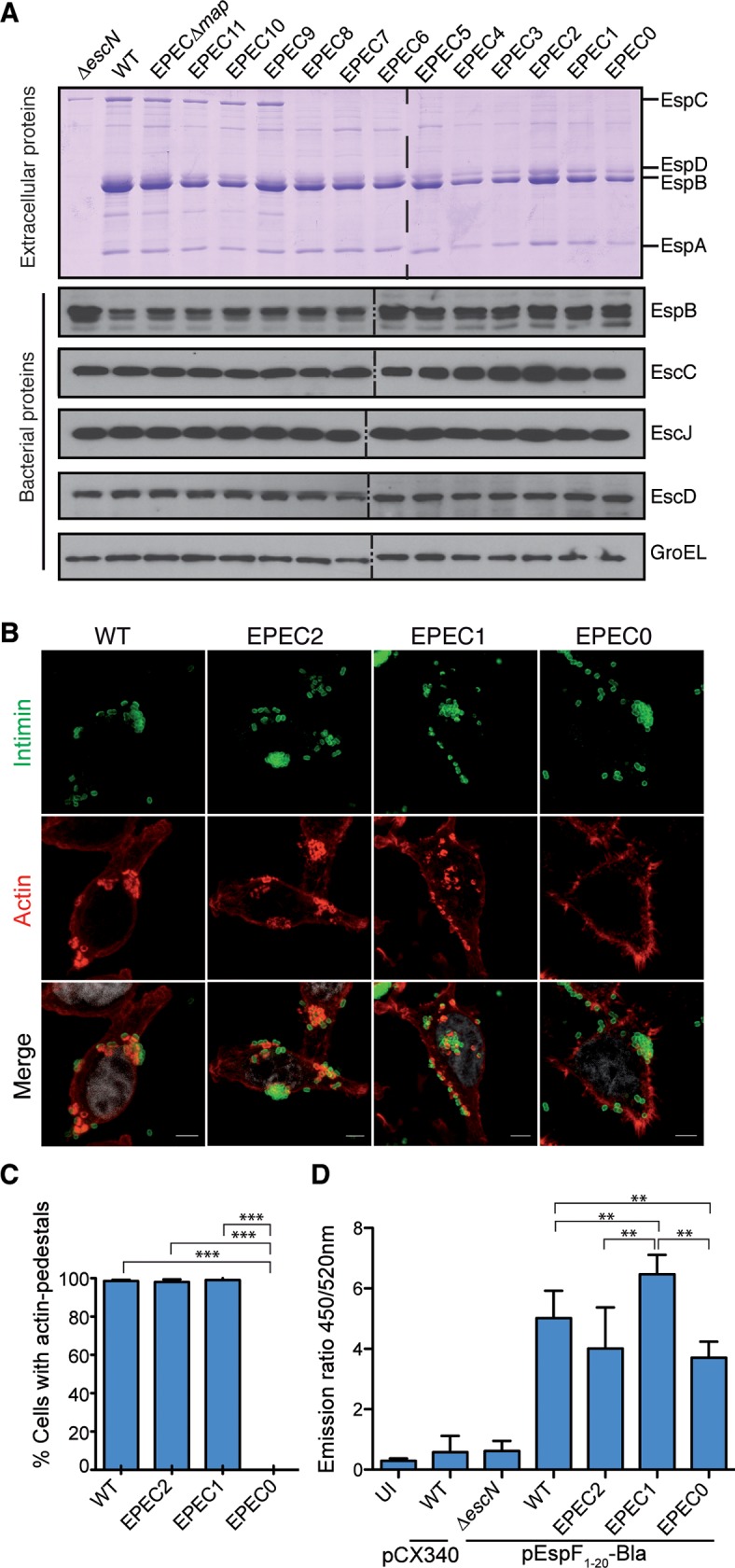
Functionality of T3SS of the EPEC effector mutants. **A**. Analysis of secreted and bacterial proteins in the indicated EPEC strains after 4 h of growth in DMEM at 37°C. Top panel: Coomassie staining of proteins found in the extracellular medium labeling the translocators EspABD and the autotransporter EspC. Molecular standards are shown in kDa. Bottom panels: Western blots of bacterial whole-cell protein extracts incubated with polyclonal antibodies to detect EscC, EscJ, EscD injectisome proteins and EspB translocator protein. Detection of cytoplasmic GroEL was used as a loading control. Discontinuous lines indicate borders of independent gels. **B**. Immunofluorescence confocal microscopy of HeLa cells infected for 90 min with WT EPEC, EPEC2, EPEC1 and EPEC0 strains. EPEC bacteria are labeled with anti-intimin-280 polyclonal (green), actin is labeled with TRITC-phalloidin (red) and cell nuclei are labeled with DAPI (gray). Actin polymerization beneath the adherent bacteria is observed in EPECwt, EPEC2 and EPEC1 strains. Scale bar 5 μm. **C.** Quantification of the number of HeLa cells with actin pedestals after infection with the indicated strains WT, EPEC2, EPEC1 and EPEC0. The data shown are the mean of two independent experiments with standard deviation (SD). In each experiment, one hundred cells per infection sample were counted. **D**. Protein translocation into HeLa cells of β-lactamase (Bla) fusion by EPEC effector mutants. HeLa cells were infected for 90 min with the indicated EPEC strains (WT EPEC, EPECΔ*escN*, EPEC2, EPEC1 EPEC0), expressing EspF_1-20_-Bla fusion (WT EPEC, EPECΔ*escN*, EPEC2, EPEC1, EPEC0) or the control vector pCX340, and then incubated with the BLA substrate CCF2/AM for additional 1 h. Bla activity was quantified measuring the emission ratio of fluorescence at 450/520 nm. Results are the mean of three independent experiments with standard deviation (SD). One way ANOVA Tukey's Multiple Comparison Test. **p<0.01 and ***p <0.001.

WT EPEC and the effector mutants EPEC2, EPEC1 and EPEC0 showed identical growth and viability at 37°C in LB and DMEM media (S5A and S5B Fig, respectively). In addition, microscopic analysis of bacteria from these cultures did not show changes in bacterial size or morphology (S5C Fig). To test the functionality of the T3SS, we analyzed the proteins secreted by WT EPEC, EPECΔ*escN* (negative control), and the effector mutant EPEC strains, after 4 h growth in DMEM at 37°C. We found that the translocators EspA, EspB and EspD, which are secreted by the T3SS [41], accumulated at roughly similar levels in the extracellular media of cultures of WT EPEC and the effector mutant strains (Fig 2A, top panel), but not in the Δ*escN* negative control. As expected, the autotransporter EspC was absent in the media of strains with deletion of IE5 (from EPEC8 to EPEC0). The expression of the structural proteins EscC, EscJ, EscD, and the translocator protein EspB, was evaluated by Western blotting in protein extracts of whole bacteria from these cultures. All the effectors mutant strains showed equal expression of the analyzed injectisome proteins compared to WT EPEC (Fig 2A, bottom panels). Detection of cytoplasmic *E*. *coli* chaperonin GroEL was used as an internal loading control. Altogether, these experiments demonstrate that the effector mutant EPEC strains are not affected in bacterial growth and express normal levels of T3SS injectisomes able to secrete the translocators.

### Tir translocation and actin pedestal formation in HeLa cells

We investigated whether the effector mutants were able to translocate Tir and trigger actin-pedestal formation upon infection of cultured mammalian cells. HeLa cells were infected with WT EPEC and the effector mutants for 1.5 h, fixed and stained for immunofluorescence microscopy. All the effector mutant strains, but EPEC0, triggered actin polymerization upon infection (Fig 2B) and form typical microcolonies, indicating the correct expression of bundle forming pili (BFP) in these strains [42]. Quantification of the number of cells with actin pedestals in these infections shows similar values for WT, EPEC2 and EPEC1, with no actin pedestals found in EPEC0 (Fig 2C).

To confirm that actin accumulation induced by EPEC2 and EPEC1 was due to intimin-Tir interaction we generated *eae* (encoding intimin) deletion mutants in both strains. Whereas EPEC2Δ*eae* and EPEC1Δ*eae* secreted normal levels of T3SS translocators (S6A Fig), they did not induce actin-pedestals in HeLa cells (S6B Fig). This demonstrates that the actin accumulations observed in HeLa cells infected by EPEC2 and EPEC1 are actual actin-pedestals caused by the specific intimin-mediated clustering of translocated Tir.

### Quantification of protein translocation by the EPEC effector mutants

We quantified the protein translocation levels of the EPEC effector mutants in HeLa cells using β-lactamase (Bla) fusions [43]. WT EPEC, EPEC2, EPEC1, EPEC0, and EPECΔ*escN* as negative control, were transformed with plasmid pEspF_1-20-_Bla, which encodes a fusion between Bla and the N-terminal 20 amino acid signal of the EspF to drive its T3SS-dependent translocation [43, 44]. WT EPEC harboring pCX340, encoding Bla without T3 signal, was used as an additional negative control. Whereas no translocation was observed with the control strains, no significant difference in the level of protein translocation was observed from WT and EPEC-2 (Fig 2D). However, EPEC1, which is devoid of *espZ*, translocated higher levels of EspF_1-20-_Bla than WT EPEC and EPEC2. Conversely, EPEC0, which lacks intimate adhesion, translocate lower levels of Bla (Fig 2D). These observations are consistent with the reported activities of EspZ and Tir [19, 35].

### Translocation of specific effectors into HeLa cells by the EPEC effector mutants

We investigated the phenotypes following translocation of selected effectors from the different EPEC mutants. In order to maintain physiological expression levels, we integrated a single copy of the effector gene of interest in its native chromosomal location. We followed the marker-less strategy for gene integration, using suicide vectors with the effector gene and flanking homology regions that preserve genome context and native regulatory elements (i.e., promoters, RBS, terminators). We integrated individually the effector genes *map* and *nleC*, into the chromosome of EPEC2, EPEC1 and EPEC0 (Table 1).

**Fig 3:**
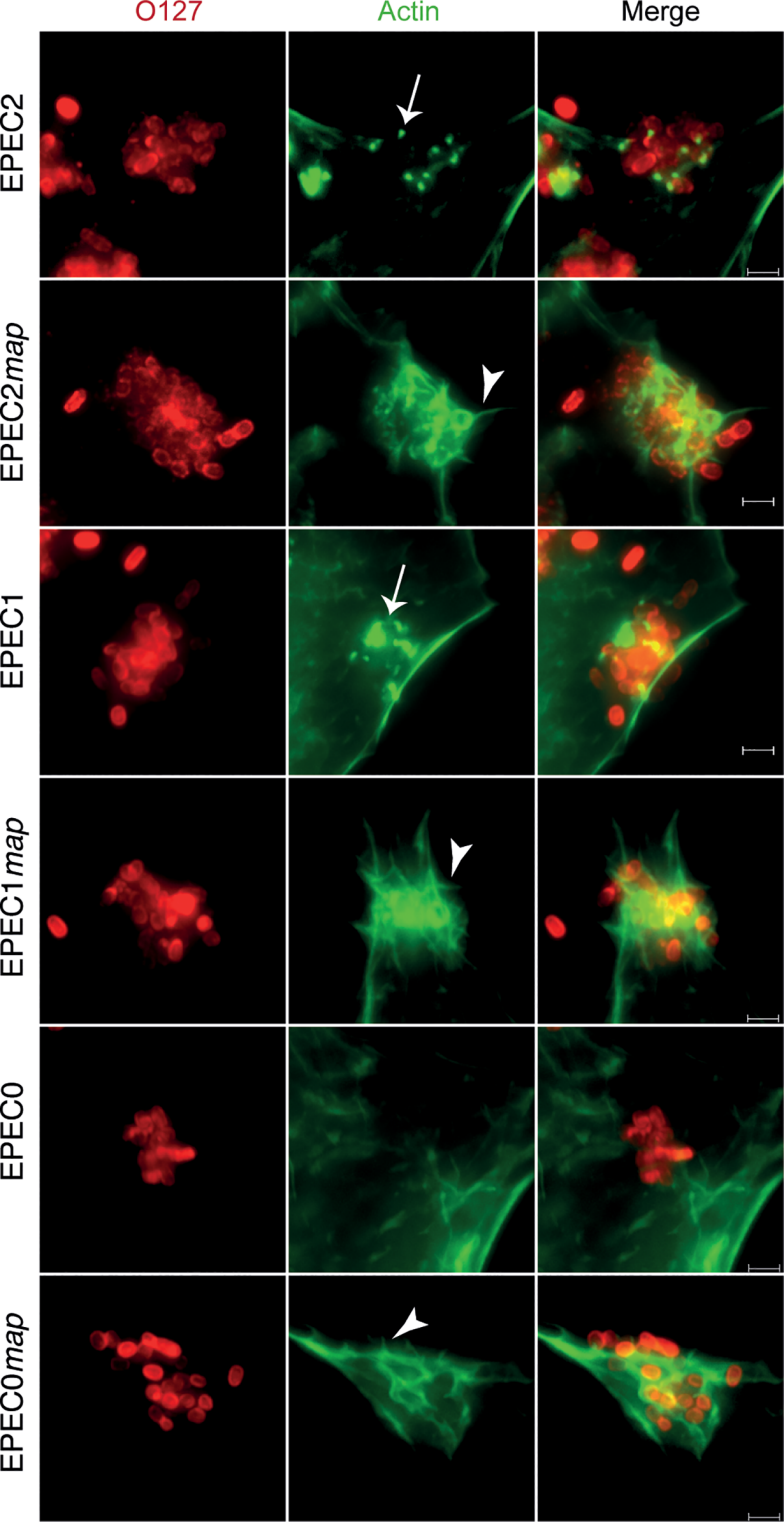
Induction of filopodia by EPEC effector mutants with integrated *map*. Immunofluorescence microscopy of Swiss 3T3 cells infected for 10 min with EPEC2, EPEC1 and EPEC0 and isogenic strains with *map* integrated in the chromosome. EPEC was detected with rabbit polyclonal anti-O127 (red) and actin was stained with Oregon-green phalloidin (green). Filopodia spikes are labelled with arrowheads. Actin accumulation beneath bacteria is indicated with arrows. Scale bar 2 μm.

We tested whether EPEC2, EPEC1 and EPEC0 expressing Map could produce filopodia early during infection. Swiss 3T3 cells were infected for 10 min with EPEC2, EPEC1 and EPEC0 and isogenic strains with an integrated copy of *map*. Actin staining of infected cells revealed the induction of filopodia by the effector mutant EPEC strains carrying *map* in the vast majority of infected cells, but not in cells infected by their parental strains (Fig 3). EPEC1+*map* showed the strongest phenotype of filopodia formation, whereas EPEC0+map induced the weakest phenotype.

**Fig 4:**
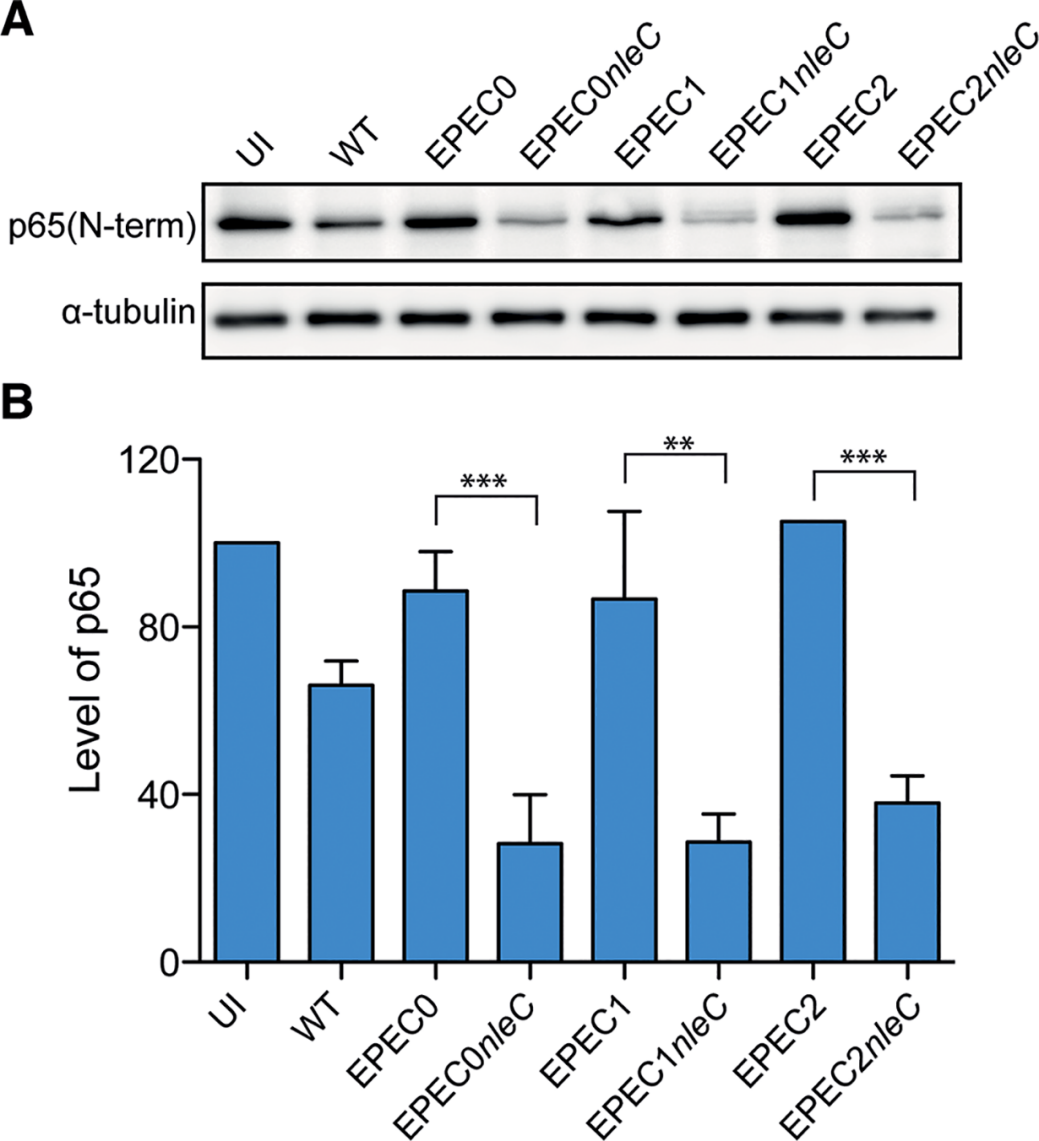
Translocation of NleC by EPEC effectors mutants induces p65 degradation. **A**. Western blot detecting NF-κB p65 protein in HeLa cells infected for 4 h (1 h+3 h gentamicin) with EPEC2, EPEC1 and EPEC0 and isogenic strains with *nleC* integrated in the chromosome. Uninfected (UI) cells and cells infected with WT EPEC are used as controls. Detection of α-tubulin was used as a loading control. **B**. Quantification of p65 in Hela cells infected with the indicated strains. Protein loading was normalized with α-tubulin. Results are the mean of three independent experiments with standard deviation (SD). One way ANOVA Tukey's Multiple Comparison Test. **p <0.01; ***p <0.001.

We next tested whether EPEC2, EPEC1 and EPEC0 expressing NleC could degrade p65. HeLa cells were infected for 4h with WT EPEC and effector mutant strains, with or without reintegrated *nleC*. Western blots of the cell lysates with anti-p65 (N-terminal) antibodies revealed that p65 was proteolysed in cells infected with all the EPEC strains expressing *nleC* (Fig 4). Proteolysis of p65 in cells infected with the effector mutant strains carrying *nleC* was higher than that induced by WT EPEC, likely caused by the presence of other effectors in EPEC (e.g. NleE, NleB) that inhibit NF-kB activation and the release of free p65 subunit, which is the preferential substrate of NleC [45–47]. Taken together, these results show that the different strains in the effector mutant library contain a functional T3SS, thus allowing us to employ them for infection of human intestinal IVOC.

### Non-LEE effectors are needed for A/E lesion formation in human IVOC

**Fig 5:**
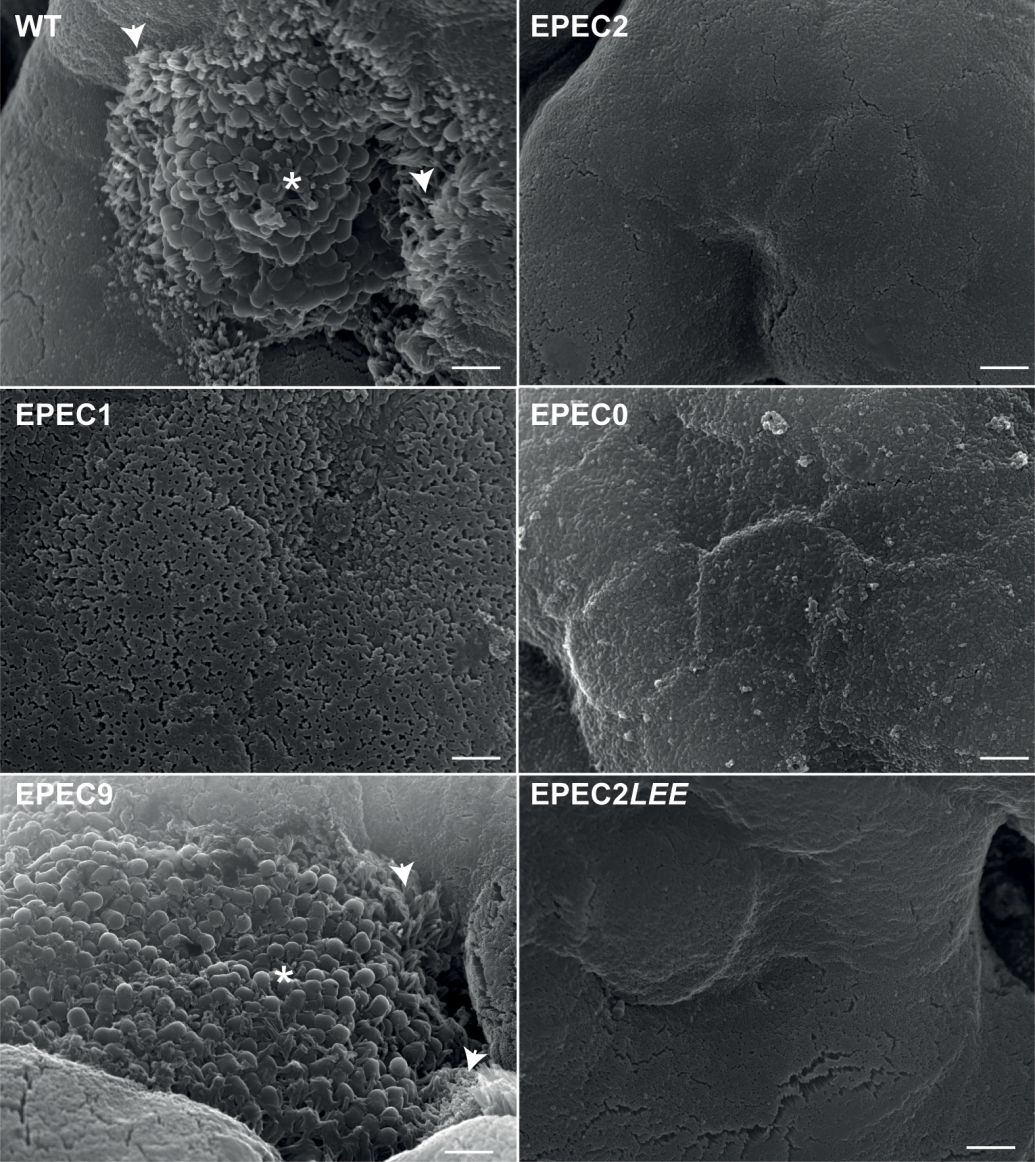
A/E lesion formation in human intestinal biopsies infected with EPEC effector mutant strains. Representative scanning electron micrographs of human duodenal biopsies infected with the indicated EPEC strains. WT EPEC and EPEC9 strains induce the characteristic A/E lesions in the intestinal mucosal surface, forming bacterial microcolonies (asterisk) and elongation of microvilli at the periphery of the microcolony (arrowheads). Biopsies infected with EPEC2, EPEC1, EPEC0 show a smooth epithelial surface without any adherent bacteria. Most biopsies infected by EPEC2*LEE* (10/11) also showed an intact mucosal surface without bacteria. Scale bar 2 μm.

We aimed to determine if intimin–Tir interaction is sufficient for A/E lesion formation on mucosal surfaces. With this in mind, we infected human duodenal biopsies with EPEC1 or EPEC2. WT EPEC and EPEC0 were used as positive and negative controls, respectively. After 7 h of infection, biopsies were washed, fixed and analyzed by scanning electron microscopy (SEM). Inspection of the mucosal surface revealed A/E lesions in ca. 77% of the biopsies infected with WT EPEC, whereas no A/E lesions were seen in IVOC infected with EPEC2, EPEC1 or EPEC0 (Fig 5 and Table 2).

**Table 2:** A/E lesions in human intestinal biopsies infected with EPEC effector mutants.

Strain	Effector genes, IEs and PPs present	A/E lesions Positive/Total (%)	*P* value *
E2348/69	wild type	17/22 (77) ^a^	
EPEC2	*espZ*, *tir*	0/6 (0)	0.0012
EPEC1	*tir*	0/6 (0)	0.0012
EPEC0	none	0/11 (0)	0.0001
EPEC2*LEE*	*espZ*, *tir*, *map*, *espH*, *espF*, *espG*	1/11 (9) ^b^	0.0005
EPEC9	*espZ*, *tir*, IE5, IE6, IE2, PP2, PP3, PP4, PP6	9/11 (82)	NS

Notes: (*) Two-tailed *P* values of Fisher’s exact test of indicated strain compared to the wild type. NS means not statistically significant (*P* values > 0.05)

^a^EPEC wt was used as a positive control in the experiments of Tables 2 and 3, which explains the higher number of biopsies infected with this strain.

^b^EPEC*LEE* formed a single A/E lesion in one biopsy.

In order to control that mucus does not affect the interaction of EPEC2 and EPEC1 with the cells and therefore the formation of A/E lesions, mucus-producing human colonic cells LS174T (S7 Fig) were infected with EPEC2, EPEC1 and EPEC0. WT EPEC was used as a positive control. This revealed similar adhesion of bacterial microcolonies and formation of actin pedestals by WT, EPEC2 and EPEC1 (S8 Fig). Hence, despite inducing actin pedestals in cultured intestinal epithelial cells *in vitro*, EPEC1 and EPEC2 could not induce A/E lesions in human intestinal tissue *ex vivo*, indicating that intimin-Tir interaction is necessary but not sufficient and that additional effectors are needed.

As the LEE is universally conserved in clinical EPEC isolates we investigated if effectors encoded on the LEE, other than Tir and EspZ, were required for A/E lesions in human intestinal biopsies. For this, we infected IVOC with a derivative strain of EPEC2, called EPEC2*LEE*, in which the genes *espG*, *map*, *espF*, and *espH* were reintegrated into their original locus on the LEE. Infection with WT EPEC was used as a control. This revealed that EPEC2*LEE* was severely impaired in its ability to form A/E lesions (Fig 5 and Table 2), as a single A/E lesion was found in only one of the eleven biopsies infected by EPEC2*LEE* (Table 2). No adherent bacteria were seen in all the other 10 IVOCs infected with EPEC2*LEE* (Fig 5). In addition, we tested whether EPEC9, which expresses all non-LEE effectors and misses all LEE effectors except EspZ and Tir, can trigger A/E lesions in IVOC. This revealed that EPEC9 induced A/E lesions in biopsies at a comparable efficiency to WT EPEC (Fig 5 and Table 2). Taken together, these results indicate that non-LEE effectors are important for A/E lesion formation by EPEC in human intestinal tissue.

### The role of Efa1/LifA homologs in A/E lesion formation *ex vivo*

To investigate the contribution of the non-LEE effectors to A/E lesion formation, we first analyzed the outcome of IVOC infection with EPEC8, EPEC7 and EPEC6. These strains are derivatives of EPEC9 having sequential deletions of effectors genes present in IE5 (*espG2*, *espC*), IE6 (*espL*, *nleB*, *nleE*, *efa1/lifA*) and IE2 (espL*, nleB*, *nleE2*, *efa1/lifA-like*) (Table 1; Fig 1). EPEC8 formed A/E lesions at the same frequency as WT and EPEC9 (Tables 2 and 3). Deletion of IE6 alone (EPEC7) caused a reduction in the frequency of A/E lesions (54%), however this did not reach significance. Moreover the proportion of biopsies with A/E lesions decreased significantly 23% following infections with EPEC6 (Table 3). Together this suggest that IE6 and IE2 contribute to A/E lesion formation. Therefore, we investigated the contribution of individual effectors found within IE2. IE2 carries the pseudogenes espL* and nleB*, as well as the effector genes *nleE2* and *efa1/lifA*-like. Therefore, we generated individual deletions of *efa1/lifA*-like and *nleE2* in EPEC7 (Table 1 and S9A Fig). We confirmed by RT-PCR that deletion of *nleE2* has no polar effects on the expression of *efa1*/*lifA*-like in IE2, and *viceversa* (S9B Fig). Infection of IVOC revealed that EPEC7Δ*nleE2* (carrying a functional copy of *efa1/lifA*-like) induced A/E lesion in 64% of the infected biopsies, similar to the parental EPEC7 strain (Table 3). In contrast, EPEC7Δ*efa1/lifA*-like triggered A/E lesions in 33% of the infected biopsies (Table 3), similar to EPEC6. These results show that deletion of *efa1/lifA-*like in EPEC7 has a significant impact on A/E lesion formation.

**Table 3:** A/E lesions formation by EPEC deletion mutants in IE effectors.

Strain	Effectors genes, IEs and PPs present	A/E lesions Positive/Total (%)	*P* value *
E2348/69	Wild type	17/22 (77)	
EPEC8	*espZ*, *tir*, IE6, IE2, PP2, PP3, PP4, PP6	10/14 (71)	NS
EPEC7	*espZ*, *tir*, IE2, PP2, PP3, PP4, PP6	7/13 (54)	NS
EPEC6	*espZ*, *tir*, PP2, PP3, PP4, PP6	3/13 (23)	0.0038
EPEC7Δ*nleE2*	*espZ*, *tir*, *lifA-like*, PP2, PP3, PP4, PP6	7/11 (64)	NS
EPEC7Δ*lifA-*like	*espZ*, *tir*, *nleE2*, PP2, PP3, PP4, PP6	4/12 (33)	0.0248
EPECΔ*lifA-*like	Wild type repertoire Δ*lifA-*like	5/6 (83)	NS
EPECΔ*lifA*	Wild type repertoire Δ*lifA*	5/5 (100)	NS
EPECΔ*lifA-*like Δ*lifA*	Wild type repertoire Δ*lifA-*like Δ*lifA*	5/6 (83)	NS
EPEC0	none	0/11 (0)	0.0001

Notes: (*) Two-tailed *P* values of Fisher’s exact test of indicated strain compared to the wild type. NS means not statistically significant (*P* values > 0.05)

To further investigate the potential role of LifA-like and LifA in EPEC A/E lesion formation, we generated single (EPECΔ*lifA-like* and EPECΔ*lifA*) and double (EPECΔ*lifA-like* Δ*lifA*) deletion mutants in WT EPEC (Table 1). These mutants secreted normal levels of EspA, EspB and EspD (S10A Fig) and produced microcolonies and actin pedestals in HeLa cells similar to the WT strain (S10B Fig). Interestingly, infection of human biopsies showed that A/E lesions were formed at efficiencies similar to the WT strain by Δ*lifA-like* and Δ*lifA* single and double mutant strains (Table 3 and S11 Fig). Collectively, these results indicate that non-LEE effectors play a major role for A/E lesion formation on human intestinal tissue *ex vivo*, and suggest an accessory role of LifA-like and LifA proteins in this process, which is masked in the presence of the entire repertoire of T3SS effectors.

## Discussion

EPEC is a major etiological agent of infant diarrhea [48, 49]. With the aim of defining the T3SS effectors implicated in A/E lesion formation, we generated a library of mutants missing part or the whole arsenal of effectors present in the prototypical strain E2348/69. We have demonstrated that the marker-less genome edition strategy generated precise deletions and gene integrations in EPEC. We have built the effector-less EPEC strain (EPEC0) devoid of all known T3SS effectors through 13 deletions, 326 bp was the smallest deletion (*espZ*) and 18260 bp the largest deletion (IE6). The effector genes were deleted from the start to the stop codon, maintaining their original transcriptional promoters and terminator signals. The only exception was the deletion of *espZ*, in which deletion of its RBS was necessary to maintain correct expression of the T3SS apparatus.

The LEE effector genes *espZ* and *tir* were deleted last as they are important to control protein translocation and bacterial attachment to host cells [19, 35, 50]. Infection of cultured epithelial cells with the WT EPEC and the effector mutant strains demonstrated the functionality of the T3SS. Infection of HeLa and mucin-producing LS174T cells with EPEC2 (*espZ* and *tir*) and EPEC1 (*tir*) showed accumulation of F-actin underneath the attached bacteria, confirming that EPEC only needs the effector Tir to induce the actin-pedestals during infection of epithelial cells *in vitro*. As expected, no pedestals were seen in cells infected with EPEC0.

Protein translocation assays indicated that all effector mutant strains, including EPEC0, translocate EspF_1-20-_Bla fusion into HeLa cells, albeit at different efficiency. EPEC1 showed the highest protein translocation level, likely due to absence of EspZ, which limits protein translocation [19]. In contrast, EPEC0 showed the lowest level of protein translocation owing to the absence of intimate adhesion [35, 50]. We have demonstrated that the EPEC effector mutants provide an excellent tool to study the function of individual effectors, under physiological expression levels, in an infection context as chromosomal single-copy integrations reproduce phenotypes previously reported for Map (filopodia formation) and NleC (NF-kB) degradation.

Importantly, using IVOC our study revealed that intimin–Tir interaction is not sufficient for A/E lesion formation and that other effector(s) are needed, as no A/E lesions were observed in biopsies infected with EPEC2 and EPEC1. Furthermore, infections of IVOC with EPEC2*LEE* (lacking all non-LEE effectors) and EPEC9 (expressing the whole non-LEE repertoire of effectors plus EspZ and Tir), showed that A/E lesion formation requires Tir and EspZ and non-LEE effectors. The difference in effector requirement for intimate adhesion of bacteria in cultured cells (Tir) and A/E lesion in intestinal tissue (Tir+non-LEE) might be due to a more stringent requirement for a productive interaction of bacteria with a complex tissue surface and/or for the hijack of cellular functions in intestinal tissue.

We further characterized the contribution of specific non-LEE effectors to A/E lesion formation by performing IVOC with mutant strains having sequential deletion of non-LEE effector genes. These experiments showed a dramatic reduction of A/E lesion formation when IE6 and, especially, IE2 are deleted (EPEC6). IE6 (*espL*, *nleB1*, *nleE1* and *efa1/lifA*) and IE2 (espL*, nleB*, *nleE2* and *efa/lifA-*like) encode a similar set of effectors. We reasoned that either *nleE2* or *efa1*/*lifA*-like effectors of IE2 should play a role in A/E lesion formation. Albeit NleE2 in IE2 has an internal deletion of 56 residues that could impede its translocation or function [51], we generated EPEC7Δ*lifA-*like and EPEC7Δ*nleE2* mutants. We found that EPEC7Δ*lifA-*like strain, but not the EPEC7Δ*nleE2* strain, exhibited a reduced efficiency of A/E lesion formation to values close to those of EPEC6, suggesting that Efa1/LifA-like protein plays a role in A/E lesion formation *ex vivo* in the effector mutants.

Efa-1/LifA-like protein was identified in the genome of EPEC E2348/69 as a homolog with aprox. 30% amino acid identity with Lymphostatin (LifA), encoded in IE6 [20]. The *lifA* gene, for lymphocyte inhibitory factor A, was first described in EPEC as a chromosomally encoded protein of 365 kDa that inhibits proliferation of lymphocytes and the synthesis of proinflammatory cytokines [26, 29]. LifA was later shown to be secreted and translocated into mammalian cells in a T3SS-dependent manner [25]. Efa-1/LifA-like homolog is also secreted in a T3-dependent manner by EPEC, but there is no evidence of its translocation into mammalian cells [25]. LifA homologs are found exclusively in the genomes of A/E pathogens[27–29]. Interestingly, *efa1/lifA* has been found physically linked to the LEE in some EHEC and EPEC strains [52]. In EHEC, EPEC, and *C*. *rodentium*, LifA/Efa-1 has been associated to cell adhesion and tissue colonization [28, 53–55]. In addition, LifA/Efa-1 proteins have been implicated in the induction of intestinal barrier disruption by manipulation of cellular Rho GTPases [56]. While playing a role in A/E lesion formation efficiency, our data show that these proteins are not essential for this process. EPEC6 and EPEC7Δ*lifA-*like strains still induce A/E lesion formation in 23–33% of infected biopsies (Table 3). Moreover, EPECΔ*lifA-*likeΔ*lifA* behaves as the WT strain forming actin-pedestals on epithelial cells *in vitro* and A/E lesions on human intestinal tissue *ex vivo*. Thus, the *efa1*/*lifA*-like proteins have an accessory role in A/E lesion formation, which is masked by other T3SS effectors found in the repertoire of the WT strain. These evidences suggest that Ea1/lifA-like protein could act in the subversion of some cellular functions needed for the establishment of the A/E lesion, but its activity can be exerted by alternative EPEC effectors found in the wild type repertoire. This fact also strengthens our experimental approach in which the role of effectors should be better analyzed in the context of infection with strains expressing a reduced and defined set of effectors, since the WT EPEC strain may have multiple effectors with overlapping, synergistic and/or antagonistic effects. The role and molecular mechanism of Efa/LifA homologs in A/E lesion formation requires further investigation.

In summary, our study shows that intimin–Tir is not sufficient for A/E lesion formation in human intestinal mucosal tissue and other effectors are needed. EPEC expressing only the LEE effectors rarely produces A/E lesions, indicating that non-LEE effectors play a major role in this process, having an additive role the effectors encoded in the IE2, IE6 and PPs.

## Methods

### Bacterial strains and growth conditions

The EPEC strains used in this work are listed in Table 1. *E*. *coli* K-12 strains used for cloning are listed in Table A of S1 Text. Bacteria were grown in Luria-Bertani (LB) liquid medium and agar-plates (1.5% w/v) or in Dulbecco's Modified Eagle Medium (DMEM), at 37 ^o^C, unless otherwise indicated. When needed for plasmid or strain selection, antibiotics were added at the following concentrations: ampicillin (Amp) at 150 μg/ml for plasmid selection, and at 75 μg/ml for selection of Amp resistance cassette in the chromosome; chloramphenicol (Cm) 30 μg/ml; kanamycin (Km) 50 μg/ml; tetracycline (Tc) 10 μg/ml; spectinomycin (Sp) 50 μg/ml. See S1 Text for details.

### Plasmids, DNA constructs, and primers

The plasmids employed in this study are listed in Table A of S1 Text. PCRs were performed with the Taq DNA polymerase (Roche, NZyTech) for standard amplifications in screenings or with the proof-reading DNA polymerases Herculase II Fusion (Agilent Technologies) or Vent DNA polymerase (NEB) for cloning purposes. When indicated, DNA was synthesized by GeneArt (Life Technologies). All DNA constructs were confirmed by DNA sequencing (Secugen and Macrogen). Oligonucleotides used in this work were obtained from Sigma and are described in Table C of S1 Text.

### EPEC genome modifications and construction of strains

A summary of genome modifications and construction of EPEC strains are listed in Table B of S1 Text. Site-specific deletions and insertions in the chromosome of EPEC were originated using a marker-less genome edition strategy with I-*Sce*I [34]. The EPEC strain to be modified was initially transformed with a plasmid pACBSR (Cm^R^) or its Sp^R^-variant pACBSR-Sp [57], both expressing the I-*Sce*I and λ-Red proteins under the control of the P_BAD_ promoter. Subsequently, these bacteria were electroporated with the corresponding pGE-based or pGETS- vector (Km^R^) and plated on LB-Km-(Cm or Sp). Selection of individual Km^R^-cointegrants and their resolution upon induction with L-arabinose for isolation of the strains with mutant alleles are described in detail in the S1 Text. All EPEC strains generated were cured of pACBSR before their analysis by serial passages on LB media lacking antibiotics and selection of Cm- or Sp-sensitive colonies. All EPEC strains were confirmed by PCR with specific primers (Tables B and C of S1 Text).

### Genome sequencing and analysis

The genomes of EPEC1 and the parental EPEC WT strains were sequenced on an Illumina Miseq platform. Average reads length between 150 and 174 bases and the global coverage was >100X. Genomes were assembled *de novo* and using reference-guided assemblies with the genome sequence of EPEC O127:H6 strain E2348/69 (nc_011601) and the *in silico*-designed reference sequence of EPEC1, as described in the S1 Text. The accession number of the genome sequence of EPEC1 effector mutant strain is <PRJEB18717>, and that of the parental EPEC WT strain E2348/69 is <PRJEB18716>. These genome sequences are available at the 'European Nucleotide Archive' http://www.ebi.ac.uk/ena/data/view/<ACCESSION.NUMBERS>.

### SDS-PAGE and Western blot

Sodium Dodecyl Sulfate–Polyacrylamide gel electrophoresis (SDS-PAGE) and Western blot were performed as reported previously [58]. Preparation of EPEC protein extracts are described in the S1 Text. For detection of EPEC proteins by Western blotting, membranes were incubated with primary rabbit antibodies anti-EspB (1:2000), anti-EscC (1:1000), anti-EscJ (1:5000), anti-EscD (1:1000) and anti-Intimin280 (1:5000). Use of polyclonal rabbit sera against EPEC Intimin-280, EscC and EscD were described previously [57, 59]. Rabbit polyclonal serum against EscJ and EspB was a kind gift of Dr. Bertha González-Pedrajo (UNAM, Mexico). Bound rabbit antibodies were detected with secondary Protein A-peroxidase (POD) conjugate (Life Technologies, 1:5000). GroEL was detected with mAb anti-GroEL-POD conjugate (1:5000; Sigma). Membranes were developed by chemiluminiscence using the Clarity Western ECL Substrate kit (Bio-Rad). The membranes were then developed by exposure to X-ray films (Agfa) or with a Fuji LAS 3000 image when the signal was quantified.

### Infection of cell cultures and fluorescence confocal microscopy

Complete description of infection conditions and microscopy is described in the S1 Text. Human HeLa cervix carcinoma cells (ATCC, CCL-2) were grown in DMEM supplemented with 10% heat-inactivated fetal bovine serum (FBS; Sigma) and 2 mM glutamine, at 37 ^o^C with 5% CO_2_. HeLa cells were washed once with pre-heated serum-free DMEM 2 h before the infection, and infected with EPEC strains for 90 min using a multiplicity of infection (MOI) of 200:1, unless indicated otherwise. Infections were stopped by three washes of sterile PBS (sigma), fixed with 4% (w/v) paraformaldehyde (in PBS, 20 min, RT) and washed again with PBS. Cells were permeabilized by incubation in a solution of 0.1% (v/v) of saponin (Sigma) in PBS for 10 min and washed with PBS. To stain EPEC strains, bacteria were incubated with polyclonal rabbit anti-intimin280 (1:500), or anti-O127 (1:100) for Δ*eae* mutants, and goat anti-rabbit secondary antibodies conjugated to Alexa488 (1:500, Life technologies) in PBS with 10% goat serum; along with Phalloidin TRITC (1:500; Sigma) and 4',6-diamidino-2-phenylindole DAPI (1:1000; Sigma) to label F-actin and DNA and mounted with 4 μl of ProLong Gold anti-fade reagent (Life technologies). To analyze translocation of NleC, HeLa cells were infected 1 h and then washed three times with PBS and incubated for additional 3 h with 200 μg/ml of gentamicin in DMEM. Cells were washed with PBS to remove unbound bacteria and cellular protein extracts were prepared analyzed by Western blot.

To analyze filopodia formation, infection with EPEC strain was done in Swiss 3T3 mouse fibroblasts (ATCC; CCL-92) cells grown in DMEM-high glucose (D5671; Sigma) supplemented with 10% of heat-inactivated fetal calf serum (FCS; Sigma), 2 mM glutamine and 1X of MEM non-essential amino acid solution 100X (Sigma). Swiss 3T3 cells were washed three times with sterile pre-warmed PBS (Sigma) and serum-free DMEM 2 h previous to the infection. Infections were done with 500 μl of EPEC cultures grown in DMEM during 3 h (aprox. MOI 500:1). The plates were centrifuged to synchronize the infection (500xg, 5 min, in a rotor pre-warmed at 37 ^o^C) and the infection was continued for additional 5 min. Infections were stopped by three washes with sterile PBS (Sigma), fixed with 4% (w/v) paraformaldehyde (in PBS, 20 min, RT) and washed with PBS. Fixed monolayers were incubated with polyclonal rabbit anti-O127 (1:100) and secondary donkey anti-rabbit-Alexa488 (Jackson ImmunoResearch, 1:100), together with Oregon-green Phalloidin (1:100, Invitrogen) to label bacteria and actin respectively. Coverslips were washed 3 times with PBS after incubation and mounted with ProLong Gold anti-fade reagent (Life technologies).

LS174T colon adenocarcinoma cells (ECACC 87060401) were grown in DMEM supplemented with 10% heat-inactivated fetal bovine serum (FBS; Sigma), 2 mM glutamine and 1X of non-essential amino acids (Sigma) at 37 ^o^C with 5% CO_2_. LS174T cells were washed three times with pre-heated PBS (Sigma) 2 h before the infection. The cells were infected with 200 μl (MOI ca. 200:1) for 90 min with EPEC grown in DMEM at 37 ^o^C as described for HeLa cells infections. Following washes with PBS (Sigma), the cells were fixed with 4% (w/v) paraformaldehyde (in PBS, 20 min, RT), washed again with PBS and permeabilized with 0.1% of Triton X-100 (Sigma) in PBS for 10 min. The immunofluorescence staining of intimin, F-actin and cell nuclei was done described previously for infections of HeLa cells. Mucin produced by LS174T cells was stained with an anti-MUC2 rabbit-polyclonal antibody (1:250 Santa Cruz biotechnology) and goat anti-rabbit IgG conjugated to Alexa488 (1:500, Life technologies) as secondary Ab.

### β-Lactamase translocation assay

β-lactamase (Bla) translocation was quantified as reported previously [43, 44] using LiveBLAzer FRET-B/G Loading Kit with CCF2-AM (ThermoFisher Scientific). Plates were read in a SpectraMax M2 fluorometer (Molecular Devices) with a filter set 450/520 nm. See S1 Text for details.

### Ethics statement

This study was performed with approval from the University of East Anglia Faculty of Medicine and Health Ethics Committee (ref 2010/11-030). All samples were registered with the Norwich Biorepository (NRES ref 08/h0304/85+5). Biopsy samples from the second part of the duodenum were obtained with informed consent during upper endoscopy of adult patients at the Norfolk and Norwich University Hospital. All samples were anonymized.

### Infection of human intestinal biopsies and scanning electron microscopy (SEM)

Up to 6 biopsy samples per donor were taken from macroscopically normal areas. Samples were cut in half and infected with EPEC wildtype and mutant strains in duplicate. Each bacterial strain was examined in human IVOC on at least three occasions using tissues from different donors. IVOC was performed as described previously [30, 60]. Briefly, biopsies were mounted on foam supports in 12 well plates and incubated with 25 μl standing overnight culture (approximately 10^7^ bacteria). Samples were incubated for 7 h on a rocking platform at 37°C in a 5% CO_2_ atmosphere. At the end of the experiment, tissues were fixed in 2.5% glutaraldehyde in PBS, dehydrated through a graded acetone series, and dried using tetramethylsilane (Sigma). Samples were blinded and examined in a scanning electron microscope (Jeol JSM-6390). Biopsies showing at least one A/E lesion were scored as positive.

### RT-PCR to evaluate *efa1/lifA*-like and *nleE2* gene expression

RNA was extracted from the EPEC strains and reversed transcribed by RT-PCR as described previously [61]. The primers used for the RT-PCR of *lifA-like*, *nleE2* and *tir* are listed in Table C of S1 Text as 108 to 113.

### Statistics

Mean and standard errors of experimental values were calculated with using Prism 5.0 (GraphPad software Inc). Statistical analyses comparing the mean of paired experimental groups were conducted with Student's t-test using Prism 5.0 (GraphPad software Inc). Statistical analyses comparing the number of A/E-positive and negative biopsies after infection with the indicated EPEC strains were conducted with Fisher's exact test to determine two-tailed *P* values using Prism 5.0 (GraphPad software Inc). Data were considered significantly different when *p*-values <0.05.

## Supporting information

S1 Text Supporting methods, tables A to D, and supplementary references.
(PDF)

S1 Fig Markerless gene deletion strategy of EPEC effector genes. Deletions were carried out by a markerless strategy using suicide plasmids with I-*Sce*I sites and mutant alleles assembled by fusing homology regions (HRs) flanking the targeted effector gene(s). Derivatives of pGE-vector contain an R6K replication origin (π protein dependent) whereas derivatives of pGETS contain a thermo-sensitive replication origin ori101 that replicates at 30°C but not at 37–42°C. The lack of π protein in EPEC, or growth at non-permissive temperature, induce integration of the suicide vector in the chromosome. Co-integrants are identified by the Km resistance phenotype. Expression of the I-*Sce*I *in vivo* from helper plasmid induces double strand brakes that are repaired by homologous recombination. Depending on the HRs involved in this second recombination, either the WT or the mutant allele can be obtained. Mutants are selected by PCR screening. Mutants do not carry any antibiotic gene marker, vector or recombination sequences ("scars").
(TIF)

S2 Fig Proteins secreted by EPEC WT and *escN* mutant generated by marker-less gene deletion strategy. **A**. Schematic representation of EPEC T3SS injectisome, indicating the filament of EspA, the translocator proteins EspB and EspD, and the ATPase EscN. Secretion of the autotransporter EspC is also shown. **B**. Coomassie staining of secreted proteins in extracellular media of EPECΔ*escN* and EPEC WT strains grown 4 h at 37°C in DMEM. The translocators EspABD and the autotransporter EspC proteins are labeled. Molecular standards mass proteins are shown in kDa.
(TIF)

S3 Fig Generation of Δ*espZ* mutant allele with wild-type levels of T3SS. **A**. Schematic representation of gene organization of *espZ* and *escI*, indicating the ribosome binding sites (RBS) in the LEE2 operon of the EPEC WT strain and Δ*espZ*-1 and Δ*espZ*-2 mutant alleles. **B**. Coomassie staining of proteins secreted in the extracellular media of EPECΔ*escN*, EPEC WT, EPECΔ*espZ*-1 and EPECΔ*espZ*-2 strains grown 4 h in DMEM at 37 ^o^C. The translocators EspABD and the autotransporter EspC are labeled. Molecular standards mass proteins are shown in kDa.
(TIF)

S4 Fig PCR to confirm deletions of effectors in EPEC0. Agarose gel electrophoresis of PCR products amplified from EPEC WT and EPEC0 strains using primers to check deletions described in Tables B and C of S1 Text. The lanes corresponding to a deletion of an effector gene or gene cluster are indicated on top. The order of the deletions is also numbered on top. DNA bands corresponding to amplicons from IE5, IE6, IE2, PP4 and PP6, in the EPECwt strain are not visible given their poor amplification due to their large size. DNA markers are labeled on the left (in bp).
(TIF)

S5 Fig Growth, viability and morphology of EPEC effector mutant strains. **A**. Growth curves of EPEC WT, EPEC2, EPEC1 and EPEC0 strains in LB following the optical density (OD) at 600nm at the indicated times points. **B**. Bacterial viability (CFU/OD_600_) of the indicated strains (EPEC WT, EPEC2, EPEC1 and EPEC0) grown in DMEM 4 h and plated in LB-agar. **C**. Immunofluorescence microscopy of bacteroa from EPEC WT, EPEC2, EPEC1 and EPEC0 strains stained with anti-intimin-280 polyclonal serum (green). Scale bar 2 μm.
(TIF)

S6 Fig Deletion of *eae* in EPEC2 and EPEC1 strains. **A**. Top panel: Coomassie staining of proteins secreted in the extracellular media by EPEC WT, EPEC2Δ*eae* and EPEC1Δ*eae* grown 4 h in DMEM at 37°C. The translocators EspABD and the autotransporter EspC are labeled. Protein standards are labelled in kilodaltons (kDa). Bottom panels: Western blots of bacterial lysates detected with rabbit polyclonal anti-intimin-280 and GroEL (as loading control). **B**. Immunofluorescence confocal microscopy of HeLa cells infected 90 min with EPEC2, EPEC2Δ*eae*, EPEC1 and EPEC1Δ*eae*. EPEC bacteria are labeled with anti-*E*. *coli* (green). Actin is labeled with TRITC phalloidin (red) and cell nuclei are labeled with DAPI (gray). Scale bar 5 μm.
(TIF)

S7 Fig Mucin-2 staining of mucus-producing LS174T cells and mucus-deficient HeLa cells. Immunofluorescence microscopy of LS174T and HeLa cells stained with anti-MUC2 rabbit-polyclonal antibody (green) and cell nuclei labeled with DAPI (gray). Scale bar 20 μm.
(TIF)

S8 Fig Infection of human intestinal LS174T cells by EPEC WT and effector mutant strains. Immunofluorescence confocal microscopy of LS174T cells infected for 90 min with WT EPEC, EPEC2, EPEC1 and EPEC0. EPEC bacteria are labeled with anti-intimin-280 polyclonal serum (green), actin is labeled with TRITC-phalloidin (red) and cell nuclei are labeled with DAPI (gray). Actin polymerization beneath the adherent bacteria is observed in WT EPEC, EPEC2 and EPEC1 strains. Scale bar 10 μm.
(TIF)

S9 Fig Deletion of *nleE2* and *efa1*/*lifA*-like in EPEC7. **A**. Clusters of effectors in IE5, IE6 and IE2. Red bars indicated deletion of IE5 and IE6 encoded-effectors and individual deletion of *nleE2* or *lifA*-like in EPEC7Δ*nleE2* and EPEC7Δ*lifA*-like, respectively. **B**. Agarose gel electropheresis of RT-PCR products of expression of *lifA-*like and *nleE2* in effectors mutant strains. EPEC7Δ*lifA-*like strain has normal expression of *nleE2*. EPEC2Δ*nleE2* has normal expression of *lifA-*like. EPEC7 has expression of *lifA-*like and *nleE2*. EPEC6 does not have expression of *lifA-*like and *nleE2*. The expression of *tir* was used as a control for RT-PCR.
(TIF)

S10 Fig Functionality of T3SS and infection of HeLa cells by deletion mutants in *efa1/lifA* and *efa1/lifA-like* genes. **A.** Coomassie staining of proteins secreted in the extracellular medium in the indicated EPEC strains grown in DMEM at 37 oC. Protein bands corresponding to the translocators EspA, EspB, EspD and the autotransporter EspC are labelled. Molecular standards are shown in kDa **B.** Immunofluorescence confocal microscopy of HeLa cells infected for 90 min with WT EPEC, EPECΔ*escN*, EPECΔ*lifA-like*, EPECΔ*lifA* and EPECΔ*lifA-like*Δ*lifA* strains. EPEC bacteria are labeled with anti-intimin-280 polyclonal serum (green), actin is labeled with TRITC-phalloidin (red) and cell nuclei are labeled with DAPI (gray). Actin polymerization beneath adherent bacteria is observed in WT EPEC, EPECΔ*lifA-like*, EPECΔ*lifA* and EPECΔ*lifA-like*Δ*lifA*. Scale bar 10 μm.
(TIF)

S11 Fig A/E lesion formation in human intestinal biopsies infected with EPEC Δ*lifA* and Δ*lifA-like* mutant strains. Scanning electron micrographs of human duodenal biopsies infected with EPECΔ*lifA*-like, EPECΔ*lifA*, and double mutant EPECΔ*lifA*-likeΔ*lifA*, showing A/E lesions formed in the intestinal mucosal surface. Large bacterial microcolonies (asterisk) and elongation of microvilli at the periphery of the microcolony (arrowheads) are labeled. Scale bar 5 μm.
(TIF)
