# Supplementary material for: Attaching and effacing (A/E) lesion formation by enteropathogenic E. coli on human intestinal mucosa is dependent on non-LEE effectors
Source: PLoS Pathog. 2017 Oct 30;13(10):e1006706. doi: 10.1371/journal.ppat.1006706 (PMC5685641; doi:10.1371/journal.ppat.1006706)
Supplement: S1 Text — Supporting methods, tables A to D, and supplementary references. (PDF) [file ppat.1006706.s001.pdf]

S1 text

# Attaching and effacing (A/E) lesion formation by enteropathogenic *E. coli* on human intestinal mucosa is dependent on non-LEE effectors


Massiel Cepeda-Molero[1], Cedric N. Berger[2], Alistair D. S. Walsham[3,4], Samuel J. Ellis[3,4], Simon Wemyss-Holden[5], Stephanie Schüller[3,4], Gad Frankel[2] and Luis Ángel Fernández[1]*

*(1) Department of Microbial Biotechnology. Centro Nacional de Biotecnología, CNB-CSIC. Darwin 3, Campus UAM, Cantoblanco, 28049 Madrid, Spain.*

*(2) MRC Centre for Molecular Bacteriology and Infection, Department of Life Sciences, Imperial College London, London, United Kingdom.*

*(3) Norwich Medical School, University of East Anglia, Norwich, UK*

*(4) Gut Health and Food Safety Programme, Quadram Institute Bioscience, Norwich, UK*

*(5) Department of Surgery, Norfolk and Norwich University Hospital NHS Foundation Trust, Norwich, UK*


***Running title***: EPEC non-LEE effectors in A/E lesion formation


***Keywords***: Attaching and effacing lesion /EPEC/ effectors/ LEE/ LifA/ type III secretion system/ human intestinal *in vitro* organ cultures (IVOC)


---


*Corresponding Author: Dr. Luis Ángel Fernández
Centro Nacional de Biotecnología, CNB- CSIC
Darwin 3, Campus UAM-Cantoblanco
Madrid 28049 (Spain).
Phone: +34 91 585 48 54
E-mail: lafdez@cnb.csic.es

## Supporting Methods

**Bacterial growth.** *E. coli* DH10B-T1$^R$ strain was used as host for the cloning and propagation of plasmids except those with R6K-origin of replication. Suicide pGE plasmids with R6K-ori were indistinctly grown in either *E. coli* strains BW25141 or CC118-λpir, which express the π protein for R6K replication [1]. Cultures of EPEC strains were grown overnight (o/n) in static at 37°C in a flask with 5 ml of liquid LB, unless otherwise indicated. Next day, cultures were diluted to a 0.05 $OD_{600}$ in 5 ml of DMEM in a well-capped Falcon tubes (BD Biosciences) and incubated in agitation (180 rpm) for 4 h for the expression of the T3SS, these cultures were used for the analysis of the T3-secreted proteins to the supernatant. For the infection of cell cultures with EPEC strains, the o/n LB cultures were diluted 1:50 in 5 ml of DMEM and grown at 37°C with 5% $CO_2$ in 50 ml Falcon tubes with the lid open in static during 2.5 or 3 h, as indicated. EPEC strains harbouring *map* and *nleC* were grown o/n with agitation at 37°C in a 30 ml tube Sterilin™ with 5 ml of liquid LB, the o/n LB bacteria cultures were diluted 1:100 in 5ml of DMEM in 30 ml tube Sterilin™ and were grown at 37°C and 5% $CO_2$, with the lid open and in static during 3 h. EPEC strains harbouring Tc$^R$-plasmids used for the β-lactamase assay were grown o/n at 37°C in LB with agitation (180 rpm) with Tc and next day were diluted to 0.05 $OD_{600}$ in 4 ml of DEMEM without Tc and grown in 6-well tissue culture plates (Falcon) during 3 h, at 37°C and 5% $CO_2$.

**Isolation of deletion and insertion mutant strains in EPEC.** The EPEC strain to be modified carried plasmid pACBSR (Cm$^R$) [2], or its Sp$^R$-variant pACBSR-Sp [3], both expressing the I-*Sce*I and λ-Red proteins under the control of the $P_{BAD}$ promoter (inducible with L-arabinose). Subsequently, bacteria were electroporated with the corresponding pGE- or pGETS- vector (Km$^R$) (Table A) and plated on LB-Km-(Cm or

Sp). Plasmids and parental strains used to obtain all the effector mutants are listed in Table B. For pGE-vectors (R6K-ori), plates were incubated at 37 °C and individual Km$^R$-colonies of the initial integration of the plasmid were grown for 6 h in LB-Cm (or Sp) liquid medium containing L-arabinose 0.4% (w/v) with agitation (180 rpm) to induce the expression of I-*Sce*I. A sample of these cultures was streaked on LB-Cm (or Sp) agar plates and incubated o/n to isolate individual colonies, which were replicated in LB-Cm (or Sp) and LB-Km-Cm (or Sp) agar plates to identify Km-sensitive colonies.

When using a pGETS-vector (pSC101-ts ori), transformants after electroporation were plated in LB-Km-Cm (or Sp) and incubated o/n at 30°C to obtain individual colonies. Several colonies were streaked on LB-Km-Cm (or Sp) plates and incubated at 42°C during 7-9 h (to induce plasmid integration), and the plates were kept o/n at 37°C. Small and large colonies grown on these plates, and the larger colonies were picked and streaked on LB-Km-Cm (or Sp) plates and incubated o/n at 37°C. Individual colonies were grown at 37°C in liquid medium containing L-arabinose and Km$^S$ colonies isolated from these cultures, exactly as described above with pGE-vectors.

In all cases, specific primers were used to identify the modified strains by PCR (Table C). Approximately, half of the Km-sensitive colonies isolated had the mutant allele in the genome while the other half contained the wild-type allele (S1 Fig.). Only in the case of the deletion of clusters of effector genes found in prophages (PPs) we utilized a positive selection to identify the mutant strains. In these cases, we performed a "double-deletion" procedure, first incorporating an Amp-FRT gene cassette with the deletion allele and selecting Amp$^R$ colonies, and later deleting this marker and selecting Amp$^S$ colonies, as follows. In the initial deletion step, the pGE or pGETS vectors had

the Amp-FRT cloned between the two homology regions (HRs) flanking the targeted gene cluster (Table A). After the initial integration of these vectors, individual colonies were grown for 6 h in LB-Amp-Cm (or Sp) liquid medium containing L-arabinose 0.4% (w/v) with agitation (180 rpm) to induce I-*Sce*I. A sample of these cultures was streaked on LB-Amp-Cm (or Sp) agar plates and incubated o/n to isolate individual colonies, which were replicated in LB-Amp-Cm (or Sp) and LB-Amp-Km-Cm (or Sp) agar plates to identify $Amp^R$ and $Km^S$ colonies, which had the deletion of the gene cluster but incorporated the Amp-FRT cassette. The Amp-FRT cassette was later deleted leaving no FRT scars by the use of pGE-vectors with the HRs flanking the targeted gene cluster but lacking the Amp-FRT cassette. After electroporation of the $Amp^R$-mutants with the corresponding pGE-vector, we followed the procedure described above for selection of cointegrants and their resolution by induction with L-arabinose. A sample of these cultures was streaked on LB-Cm (or Sp) agar plates and incubated o/n to isolate individual colonies, which were replicated in LB-Cm (or Sp), LB-Amp-Cm (or Sp) and LB-Km-Cm (or Sp) agar plates to identify colonies that were both Amp-sensitive and Km-sensitive. Deletion of the effector gene cluster and the Amp-FRT cassette was confirmed with specific primers by PCR.

**Genome sequencing and analysis.** A single colony of the bacterial strain EPEC1 or EPECwt was inoculated in 5 ml of LB and grown with shaking o/n at 37°C. Bacteria were harvested and the genomic DNA was using GNOME® DNA Isolation Kit (MP Biomedicals). The DNA libraries were prepared with NEBNext Ultra DNA Library Prep Kit (New England BioLabs). The library was sequenced on an Illumina Miseq platform. After quality filtering 97.56% and 98.09% of the total number of reads were high quality, in EPECwt and EPEC1 respectively, with an average read length of 150

bases in EPECwt and 174 bases in EPEC1. The reads obtained were quality filtered using NGS QC Toolkit [4] and used to build a reference-guided assembly with BWA [5] against the EPEC O127:H6 strain E2348/69 (nc_011601) and the *in silico* designed reference sequence of EPEC1, followed by processing of the results with Samtools [6]. Mean coverage for EPEC1 was 112X and for EPEC WT 210X. *De novo* assemblies of the quality-filtered reads were also carried out using the A5 assembler [7]. The assemblies were visualized with IGV [8]. Genome annotation was carried out using RATT [9]. All analysis and annotation steps were automated using in-house developed scripts (Scientific computing service of CNB-CSIC). Genome comparisons were carried out using the Last aligner [10] and the Mummer toolkit [11]. Differences between EPECwt (PRJEB18716) and EPEC1 (PRJEB18717) genome are listed in Table D. Sequence comparison with EPEC O127:H6 strain E2348/69 (nc_011601) confirmed a perfect match with EPECwt except for the presence of four gaps: gap1 between bp positions 1267984 and 1308771; gap2 between bp positions 3221322 and 3243834; gap3 between bp positions 3367950 and 3420209; gap4 between bp positions 4461321 and 4462695. Positions referred to sequence nc_011601.

**Protein extracts.** For the analysis of proteins in EPEC, bacteria were harvested from 1 ml aliquots of the cultures by centrifugation (14000xg, 5 min). To obtain whole-cell protein extracts, the cells were resuspended in 400 μl of phosphate-buffered saline (PBS), mixed with 100 μl of 5X SDS-PAGE sample buffer and boiled for 10 min, and then protein were separated by SDS-PAGE and analyzed by Western blot. For the analysis of secreted proteins, the bacterial cultures were centrifuged 5 min at 4000 rpm (4 °C) in 50 ml Falcon tubes (BD Biosciences). Culture supernatant was collected centrifuged again under the same conditions to ensure removal of bacteria. Later 1 ml of the culture supernatants was chilled on ice and incubated 60 min with trichloroacetic

acid (TCA 20% w/v; Merck) for protein precipitation. After centrifugation (14000xg, 15 min, 4 °C), TCA-precipitated protein pellets were rinsed with cold acetone (-20 ºC) and resuspended in 30 µl of SDS-PAGE sample buffer for Coomassie staining.

To evaluate the amount p65 protein into HeLa cells after infection with EPEC strains. Infected HeLa cells (aprox. $1.2x10^6$ cells) were scraped from one well of a 6-well tissue culture plate (Falcon) using 500 µl of lysis buffer (1X). Lysis buffer 1X was prepared by with 175 µl of Tetrasodium Diphosphate Decahydrate-NaPPi 250 mM, 25 µl of Protease Inhibitor Cocktail (P8340, Sigma), 1 ml of lysis buffer (7X) and 5.80 ml of $H_2O$. The lysis buffer 7X is: 350 mM Tris-base (pH 7.4), 1.05 M NaCl, 14 mM EDTA, and 7%(v/v) NP-40. This buffer was kept in aliquots of 1 ml at -20 °C until use. The lysed cell samples were recovered in a 1.5 ml tube and incubated during 30 min at 4 °C with rotation. Then samples were centrifuged (14000xg, 15 min) and 400 µl of the supernatant were mixed with 5X SDS-PAGE sample buffer and then protein were separated by SDS-PAGE and analyzed by Western blot. The membranes were incubated with rabbit polyclonal anti-NF-κB p65 (SC-109 Santa Cruz, 1:1000) and mouse monoclonal anti-alpha Tubulin (clone DM1A Sigma, 1:1000). Bound antibodies were detected with goat anti-rabbit POD (Jackson ImmunoResearch, 1:5000) and goat anti-mouse POD (Jackson ImmunoResearch, 1:10000) respectively.

**Infection of cell cultures and fluorescence confocal microscopy.** The human cell lines HeLa (ATCC, CCL-2) were routinely grown as monolayers in DMEM supplemented with 10% heat-inactivated fetal bovine serum (FBS; Sigma) and 2 mM glutamine, at 37 °C with 5% $CO_2$. HeLa cells were seeded on 24-well tissue culture plates (Falcon) with sterile coverslips approximately 24 h before the infection to obtain ~90% cell confluence (i.e. $2x10^5$ cells/well). HeLa cells were washed once with pre-heated serum-free DMEM 2 h before the infection, and infected with EPEC strains for

90 min using a multiplicity of infection (MOI) of 200:1, unless indicated otherwise. Infections were stopped by three washes of sterile PBS (sigma), fixed with 4% (w/v) paraformaldehyde (in PBS, 20 min, RT) and washed again with PBS three times. Cells were permeabilized by incubation in a solution of 0.1% (v/v) of saponin (Sigma) in PBS for 10 min and washed with PBS three times. To stain EPEC strains, bacteria were incubated with polyclonal rabbit anti-intimin280 (1:500), or anti-O127 (1:100; a gift of Dr. Jorge Blanco, Universidad Santiago de Compostela) for Δ*eae* mutants, in PBS with 10% (v/v) goat serum (Sigma) for 60 min at RT. For mucin staining cells were incubated with polyclonal rabbit anti-MUC2 (1:250 Santa Cruz) for 60 min RT. Coverslips were washed three times with PBS and incubated 45 min at RT with goat anti-rabbit secondary antibodies conjugated to Alexa488 (1:500, Life technologies) in PBS with 10% goat serum; along with Phalloidin TRITC (1:500; Sigma) and 4',6-diamidino-2-phenylindole DAPI (1:1000; Sigma) to label F-actin and DNA, respectively. Coverslips were washed 3 times with PBS and were mounted with 4 µl of ProLong Gold anti-fade reagent (Life technologies). Mounted coverslips were dried o/n at RT. Samples were observed in a SP5 confocal microscope (Leica) using the 100X objective or a fluorescence Olympus microscope (BX61) using the 40X and 60X objective.

To analyze the phenotype of filopodia formation, infection with EPEC strain was done in Swiss 3T3 mouse fibroblasts (ATCC; CCL-92) cells because these cells have more active actin dynamic than HeLa cells. Swiss 3T3 were grown as monolayer in DMEM-high glucose, with 4500 mg glucose/l (D5671; Sigma) supplemented with 10% of heat-inactivated fetal calf serum (FCS; Sigma), 2 mM glutamine and 1X of MEM non-essential amino acid solution 100X (Sigma). The Swiss 3T3 cells were seeded on 24-well tissue culture plates (Falcon) with coverslips 48 h before the infection to obtain cell

confluence between 60-70% (ca. 1.3x10$^5$ cells). Swiss 3T3 cells were washed three times with sterile pre-warmed PBS (Sigma) and serum-free DMEM 2 h previous to the infection. Infections were done with 500 µl of EPEC cultures grown in DMEM during 3 h (aprox. MOI 500:1). The plates were centrifuged to synchronize the infection (500xg, 5 min, in a rotor pre-warmed at 37 °C) and the infection was continued for additional 5 min. Infections were stopped by three washes with sterile PBS (Sigma), fixed with 4% (w/v) paraformaldehyde (in PBS, 20 min, RT) and washed again with PBS three times. Fixed monolayers were treated with 50 mM $NH_4Cl$ (10 min, RT) for formaldehyde neutralization, permeabilized by incubation with 0.2% (v/v) Triton X-100 in PBS (4 min, RT), and washed with PBS twice. Coverslips were treated with PBS-0.2% BSA (10 min, RT) and incubated with polyclonal rabbit anti-O127 (1:100) in PBS-0.2% BSA for 60 min at RT to stain EPEC bacteria. Coverslips were washed twice with PBS and then treated with PBS-0.2% BSA during 5 min. Then coverslips were incubated 45 min with donkey anti-rabbit-Alexa488 (Jackson ImmunoResearch, 1:100) in PBS-0.2% BSA together with Oregon-green Phalloidin (1:100, Invitrogen) to label bacteria and actin respectively. Coverslips were washed 3 times with PBS after incubation and mounted with ProLong Gold anti-fade reagent (Life technologies). Samples were observed at the Zeiss Axio imager microscope. The projections of the images were done with AxioVision LE Software (Zeiss).

To analyze translocation of NleC, HeLa cells were grown in DMEM supplemented with 10% fetal bovine serum (FBS; Sigma), 2 mM glutamine and 1% of MEM non-essential amino acid solution 100X (Sigma). HeLa cells were seeded on 6-well tissue culture plates (Falcon) 48 h before to the infection to obtain cell confluence between 70% and 80% (aprox. 1.2x10$^6$ cells). Previous to the infection, HeLa cells were washed three times with sterile pre-warmed PBS (Sigma) and serum-free DMEM was added for 2 h.

Infections were done with 1.5 ml of EPEC cultures (MOI 200:1), except for EPEC0 strains in which 3 ml were utilized (MOI 400:1), grown in DMEM 3 h previous to the infection. Cells were infected 1 h and then washed three times with PBS and incubated for additional 3 h with 200 μg/ml of gentamicin in DMEM. Cells were washed with PBS to remove unbound bacteria and cellular protein extracts were prepared analyzed by Western blot (see above).

The adenocarcinoma colon human cells LS174T (ECACC 87060401) were routinely grown in DMEM supplemented with 10% heat-inactivated fetal bovine serum (FBS; Sigma) and 2 mM glutamine and 1X non essential amino acids (Sigma) at 37 °C with 5% $CO_2$. LS174T cells were seeded at a density of $10^5$ cells per well on 24-well tissue culture plates (Falcon) with sterile coverslips for 7 days to reach 100% cell confluence. LS174T cells were washed three times with pre-heated PBS (Sigma) 2 h before the infection and serum-free DMEM was added. Infection was done using 200μl of preactivated EPEC strains during 90 min. Infections were stopped by three washes of sterile PBS (Sigma), fixed with 4% (w/v) paraformaldehyde (in PBS, 20 min, RT) and washed again with PBS three times. Cells were permeabilized by incubation in a solution of 0.1% of Triton X-100 (Sigma) in PBS for 10 min and washed with PBS three times. The immunofluorescence staining was done as described previously for HeLa cells.

**β-Lactamase translocation assay.** HeLa cells were seeded in a 96-well opaque plate (Nunc) 48 h previous to the infection to obtain a cell confluence of $4x10^4$ cell/well, at 37°C with 5% $CO_2$.The culture medium of the cells was changed 2 h before to the infection by serum-free DMEM. HeLa cells were infected with the induced EPEC strains with a MOI of 100:1, after 30 min of infection 1 mM of isopropyl β-D-1-thiogalactopyranoside IPTG was added, and the infections were maintained during 1

extra hour under these conditions. Then cells were washed twice with pre-heated DMEM and cover with 100 µl of Hanks' balanced salt solution (HBSS; Sigma) and 20 µl of 6X CCF2/AM solution (final concentration 1 µM) following manufacturer instructions (LiveBLAzer™ FRET-B/G Loading Kit with CCF2-AM; ThermoFisher Scientific). Samples were incubated for 1 h in the dark at a room temperature (RT) and plates were read in a SpectraMax M2 fluorometer (Molecular Devices) with a filter set 450/520 nm.

**Plasmid constructs**. Plasmids based on pGE- or pGETS-vector backbones were constructed for the deletion and insertion of effector genes sequences in the chromosome of EPEC (Table A). A list of all EPEC mutants generated indicating parental strain and plasmid used can be found in Table B. Oligonucleotide primers used in DNA constructs are described in Table C. Vectors pGE [12], pGETS [3], pACBSR [2] and pACBSR-Sp [3] have been described previously.

pGETSΔ*map*: is a pGETS-derivate with two homology regions (HRs) of 300 bp flanking *map* ORF of EPEC, the HRs are fused and were synthesized (GeneArt, Life Technologies) with flanking *Xho*I-*Sph*I restriction sites and cloned into the same sites of pGETS.

pGEΔ*espG*: is a pGE-derivate with two HRs of ca. 700 bp flanking the *espG* ORF that were amplified by PCR from the chromosome of EPEC. The HR1 was amplified with primers 3 and 4 and the HR2 was amplified with primers 5 and 6. The HRs were fused by a fusion PCR using primers 3 and 6 and the resulting DNA fragment was cloned with *Xho*I and *Sph*I in the pGE-backbone.

pGETSΔ*espF*: is a pGETS-derivate with two homology regions (HRs) of 300 bp flanking the *espF* ORF of EPEC, the HRs are fused and were synthesized (GeneArt,

Life Technologies) with flanking *Xho*I-*Sph*I restriction sites and cloned into the pGETS-backbone.

pGEΔ*espH*: is a pGE-derivate with two HRs of ca. 600 bp flanking the *espH* gene that were amplified by PCR from the chromosome of EPEC. The HR1 was amplified with primers 11 and 12 and the HR2 was amplified with primers 13 and 14. The HRs were fused by a fusion PCR using primers 11 and 14 and the resulting DNA fragment was cloned with *Xho*I and *Sph*I in the pGE-backbone.

pGEΔIE5: is a pGE-derivate with two HRs of ca. 600 bp flanking the cluster of effector genes in IE5 (*espC* and *espG*) that were amplified by PCR from the chromosome of EPEC. The HR1 was amplified with primers 15 and 16 and the HR2 was amplified with primers 17 and 18. The HR1 was digested *Xho*I-*Sac*I, the HR2 was digested *Sac*I-*Sph*I and the pGE-vector was digested *Xho*I-*Sph*I. The HRs were cloned with a triple ligation between *Xho*I-*Sac*I-*Sph*I sites in the pGE-backbone.

pGEΔIE6: is a pGE-derivate with two HRs (HR1 1000 bp and HR2 ca. 600 bp) flanking the cluster of effector genes in IE6 (*espL*, *nleB*, *nleE*, e*fa1/lifA*) that were amplified by PCR from the chromosome of EPEC. The HR1 was amplified with primers 21 and 22 and the HR2 was amplified with primers 23 and 24. The HR1 was digested *Xho*I-*Sac*I, the HR2 was digested *Sac*I-*Sph*I and the pGE-vector was digested *Xho*I-*Sph*I. The HRs were cloned with a triple ligation between *Xho*I-*Sac*I-*Sph*I sites in the pGE-backbone.

pGEΔIE2: is a pGE-derivate with two HRs (HR1 1000 bp and HR2 ca. 600 bp) flanking the cluster of effector genes in IE2 (*efa1/lifA, nleE, nleB**, *espL**) that were amplified by PCR from the chromosome of EPEC. The HR1 was amplified with primers 27 and 28 and the HR2 was amplified with primers 29 and 30. The HR1 was digested *Xho*I-*Sac*I, the HR2 was digested *Sac*I-*Spe*I and the pGE-vector was digested

*Xho*I-*Spe*I. The HRs were cloned with a triple ligation between *Xho*I-*Sac*I-*Spe*I sites in the pGE-backbone.

pGEΔPP2: is a pGE-derivate with two HRs (HR1 300 bp and HR2 ca. 700 bp) flanking the cluster of effector genes in PP2 (*nleH, Cif*, espJ*) that were amplified by PCR from the chromosome of EPEC. The HR1 was amplified with primers 33 and 34 and the HR2 was amplified with primers 35 and 36. The HR1 was digested *Xho*I-*Sac*I, the HR2 was digested *Sac*I-*Sph*I and the pGE-vector was digested *Xho*I-*Sph*I. The HRs were cloned with a triple ligation between *Xho*I-*Sac*I-*Sph*I sites in the pGE-backbone.

pGEΔPP2-Amp-FRT: is a pGEΔPP2-derivate with the *bla* gene ($Amp^R$) flanked by two FRT sites inserted between the HRs. The Amp-FRT cassette flanked by *Sac*I restriction sites was amplified with primers 57 and 59 from plasmid pGE-Amp-FRT and cloned in the *Sac*I restriction site of the pGEΔPP2. Plasmid pGE-Amp-FRT was constructed by amplification of the Ampicillin resistance gene from pUC18 [13] with oligos incorporating FRT sites (Table C).

pGEΔPP3: is a pGE-derivate with two HRs (HR1 ca. 400 bp and HR2 ca. 600 bp) flanking *nleJ* gene that were amplified by PCR from PP3 in the chromosome of EPEC. The HR1 was amplified with primers 39 and 40 and the HR2 was amplified with primers 41 and 42. The HR1 was digested *Xho*I-*Sac*I, the HR2 was digested *Sac*I-*Sph*I and the pGE-vector was digested *Xho*I-*Sph*I. The HRs were cloned with a triple ligation between *Xho*I-*Sac*I-*Sph*I in the pGE-backbone.

pGEΔPP3-Amp-FRT: is a pGEΔPP3-derivate with the *bla* gene ($Amp^R$) flanked by two FRT sites inserted between the HRs. The Amp-FRT cassette flanked by *Sac*I restriction sites was amplified with primers 57 and 59 from a plasmid carrying *bla* gene, and cloned in the *Sac*I restriction site of the pGEΔPP3.

pGEΔPP4: is a pGE-derivate with two HRs (HR1 300 bp and HR2 ca. 900 bp) flanking the cluster of effector genes in PP4 (*nleG, nleB, nleC, nleH*, nleD*) that were amplified by PCR from the chromosome of EPEC. The HR1 was amplified with primers 45 and 46 and the HR2 was amplified with primers 47 and 48. The HR1 was digested *Xho*I-*Sac*I, the HR2 was digested *Sac*I-*Sph*I and the pGE-vector was digested *Xho*I-*Sph*I. The HRs were cloned with a triple ligation between *Xho*I-*Sac*I-*Sph*I in the pGE-backbone.

pGEΔPP4-Amp-FRT: is a pGEΔPP4-derivate with the *bla* gene ($Amp^R$) flanked by two FRT sites inserted between the HRs. The Amp-FRT cassette flanked by *Sac*I restriction sites was amplified with primers 57 and 59 from plasmid carrying the *bla* gene and cloned in the *Sac*I restriction site of pGEΔPP4.

pGEΔPP6: is a pGE-derivate with two HRs of ca. 500 bp flanking the cluster of effector genes in PP6 (*nleA/espL, nleH ,nleF, espO*) that were amplified by PCR from the chromosome of EPEC. The HR1 was amplified with primers 51 and 52 and the HR2 was amplified with primers 53 and 54. The HR1 was digested *Xho*I-*Sac*I, the HR2 was digested *Sac*I-*Sph*I and the pGE-vector was digested *Xho*I-*Sph*I. The HRs were cloned with a triple ligation between *Xho*I-*Sac*I-*Sph*I in the pGE-backbone.

pGETSΔPP6-Amp-FRT: is a pGETS-derivate with two HRs of ca. 500 bp flanking the cluster of effector genes in PP6 (*nleA/espL, nleH ,nleF, espO*). The pGEΔPP6 was digested *Xho*I-*Sph*I and the DNA fragment with the HRs was cloned in the pGETS backbone. The Amp-FRT cassette flanked by *Sac*I restriction sites was amplified with primers 58 and 59 from a plasmid carrying the *bla* gene and cloned in the *Sac*I restriction site of pGETSΔPP6.

pGEΔ*espZ*-2: is a pGE-derivate with two HRs of ca. 700 bp flaking the *espZ* gene that were amplified by PCR from EPEC chromosome. The HR1 was amplified with primers 71 and 72 and the HR2 was amplified with primers 73 and 74. The HR1 was digested

*Xho*I-*Sac*I, the HR2 was digested *Sac*I-*Sph*I and the pGE-vector was digested *Xho*I-*Sph*I. The HRs were cloned with a triple ligation between *Xho*I-*Sac*I-*Sph*I in the pGE-backbone.

pGE$\Delta$*tir*: is a pGE-derivate with two HRs (HR1 300 bp and HR2 ca. 700 bp) flaking the *tir* gene that were amplified by PCR from EPEC chromosome. The HR1 was amplified with primers 77 and 78 and the HR2 was amplified with primers 79 and 80. The HR1 was digested *Xho*I-*Sac*I, the HR2 was digested *Sac*I-*Sph*I and the pGE-vector was digested *Xho*I-*Sph*I. The HRs were cloned with a triple ligation between *Xho*I-*Sac*I-*Sph*I in the pGE-backbone.

pGE$\Delta$*escN*: is a pGE-derivate with two HRs of ca. 600 bp flaking the *escN* gene that were amplified by PCR from EPEC chromosome. The HR1 was amplified with primers 60 and 61 and the HR2 was amplified with primers 62 and 63. The HR1 was digested *Xho*I-*Sac*I, the HR2 was digested *Sac*I-*Sph*I and the pGE-vector was digested *Xho*I-*Sph*I. The HRs were cloned with a triple ligation between *Xho*I-*Sac*I-*Sph*I in the pGE-backbone.

pGE$\Delta$*eae*: is a pGE-derivate with two HRs of ca. 500 bp flanking *eae* gene. The HRs were amplified by PCR from EPEC chromosome. The HR1 was amplified with primers 102 and 103 and the HR2 was amplified with primers 104 and 105. The HR1 was digested *Xho*I-*Sac*I, the HR2 was digested *Sac*I-*Sph*I and the pGE-vector was digested *Xho*I-*Sph*I. The HRs were cloned with a triple ligation between *Xho*I-*Sac*I-*Sph*I in the pGE-backbone.

pGE+*map*: is pGE-derivatibe with the *map* gene and flanking HRs as in the chromosome of EPEC. The DNA fragment with *map* gene plus the HRs was amplified with primers 66 and 67 from EPEC chromosome and was cloned *Xho*I-*Sph*I in the pGE-backbone.

pGE+*espH*: is pGE-derivate with the *espH* gene and flanking HRs as in the chromosome of EPEC. The DNA fragment with *espH* gene plus the HRs was amplified with primers 11 and 68 from EPEC chromosome and was cloned *Xho*I-*Spe*I in the pGE-backbone.

pGE+*espF*: is a pGE-derivate with the *espF* gene and flanking HRs as in the chromosome of EPEC. The DNA fragment with *espF* gene plus the HRs was amplified with primers 69 and 70 from EPEC chromosome and was cloned *Xho*I-*Sph*I in the pGE-backbone.

pGE+*espG*: is pGE-derivate with the *espG* gene plus the HRs as in the chromosome of EPEC. The DNA fragment with *espG* gene plus the HRs was amplified with primers 3 and 6 from EPEC chromosome and was cloned *Xho*I-*Sph*I in the pGE-backbone.

pGEΔPP4+*nleC*: is a pGEΔPP4-derivate with the intergenic region between *nleG* and *nleB* fused to *nleC* plus the HRs as in the chromosome of EPEC. The intergenic region between *nleG* and *nleB2* was amplified with primers 83 and 84 and the *nleC* gene was amplified with primers 85 and 86 from EPEC chromosome. Both fragments were fused by a fusion PCR using primers 83 and 86 and the resulting DNA fragment was cloned in the *Sac*I site between the HRs in the pGEΔPP4.

pGEΔ*lifA*-like: is a pGEΔIE2-derivative with HRs flanking *lifA-like* gene in IE2 of EPEC. The HR2 was amplified with primers 87 and 88 from EPEC chromosome and was cloned between *Sac*I-*Sph*I sites in the pGEΔIE2.

pGEΔ*lifA*: is a pGEΔIE6-derivative with HRs flanking *lifA* gene in IE6 of EPEC. The HR1 was amplified with primers 114 and 115 from EPEC chromosome and was cloned between *Xho*I-*Sac*I sites in the pGEΔIE6.

pGEΔ*nleE2*: is a pGE-derivate with two HRs of ca. 500 bp flanking *nleE2* gene. The HRs were amplified by PCR from IE2 in the chromosome of EPEC. The HR1 was

amplified with primers 90 and 91 and the HR2 was amplified with primers 92 and 93. The HR1 was digested *Xho*I-*Sac*I, the HR2 was digested *Sac*I-*Sph*I and the pGE-vector was digested *Xho*I-*Sph*I. The HRs were cloned with a triple ligation between *Xho*I-*Sac*I-*Sph*I in the pGE-backbone.

**Supplementary References**


1. Stalker DM, Kolter R, Helinski DR. Plasmid R6K DNA replication. I. Complete nucleotide sequence of an autonomously replicating segment. J Mol Biol. 1982;161(1):33-43. Epub 1982/10/15. PubMed PMID: 6759660.
2. Herring CD, Glasner JD, Blattner FR. Gene replacement without selection: regulated suppression of amber mutations in *Escherichia coli*. Gene. 2003;311:153-63. Epub 2003/07/11. doi: S0378111903005857 [pii]. PubMed PMID: 12853150.
3. Ruano-Gallego D, Álvarez B, Fernández LÁ. Engineering the Controlled Assembly of Filamentous Injectisomes in *E. coli* K-12 for Protein Translocation into Mammalian Cells. ACS synthetic biology. 2015;4(9):1030-41. doi: 10.1021/acssynbio.5b00080.
4. Patel RK, Jain M. NGS QC Toolkit: a toolkit for quality control of next generation sequencing data. PLoS One. 2012;7(2):e30619. doi: 10.1371/journal.pone.0030619. PubMed PMID: 22312429; PubMed Central PMCID: PMCPMC3270013.
5. Li H, Durbin R. Fast and accurate short read alignment with Burrows-Wheeler transform. Bioinformatics. 2009;25(14):1754-60. doi: 10.1093/bioinformatics/btp324. PubMed PMID: 19451168; PubMed Central PMCID: PMCPMC2705234.
6. Li H, Handsaker B, Wysoker A, Fennell T, Ruan J, Homer N, et al. The Sequence Alignment/Map format and SAMtools. Bioinformatics. 2009;25(16):2078-9. doi: 10.1093/bioinformatics/btp352. PubMed PMID: 19505943; PubMed Central PMCID: PMCPMC2723002.
7. Tritt A, Eisen JA, Facciotti MT, Darling AE. An integrated pipeline for de novo assembly of microbial genomes. PLoS One. 2012;7(9):e42304. doi: 10.1371/journal.pone.0042304. PubMed PMID: 23028432; PubMed Central PMCID: PMCPMC3441570.

8. Thorvaldsdottir H, Robinson JT, Mesirov JP. Integrative Genomics Viewer (IGV): high-performance genomics data visualization and exploration. Brief Bioinform. 2013;14(2):178-92. doi: 10.1093/bib/bbs017. PubMed PMID: 22517427; PubMed Central PMCID: PMCPMC3603213.

9. Otto TD, Dillon GP, Degrave WS, Berriman M. RATT: Rapid Annotation Transfer Tool. Nucleic Acids Res. 2011;39(9):e57. doi: 10.1093/nar/gkq1268. PubMed PMID: 21306991; PubMed Central PMCID: PMCPMC3089447.

10. Kielbasa SM, Wan R, Sato K, Horton P, Frith MC. Adaptive seeds tame genomic sequence comparison. Genome Res. 2011;21(3):487-93. doi: 10.1101/gr.113985.110. PubMed PMID: 21209072; PubMed Central PMCID: PMCPMC3044862.

11. Delcher AL, Phillippy A, Carlton J, Salzberg SL. Fast algorithms for large-scale genome alignment and comparison. Nucleic Acids Res. 2002;30(11):2478-83. PubMed PMID: 12034836; PubMed Central PMCID: PMCPMC117189.

12. Piñero-Lambea C, Bodelón G, Fernández-Periáñez R, Cuesta AM, Álvarez-Vallina L, Fernández LA. Programming Controlled Adhesion of *E. coli* to Target Surfaces, Cells, and Tumors with Synthetic Adhesins. ACS synthetic biology. 2015;4(4):463-73. Epub 2014/07/22. doi: 10.1021/sb500252a. PubMed PMID: 25045780.

13. Viera J, Messing J. The pUC plasmids, an M13mp7-derived system for insertion mutagenesis and sequencing with synthetic universal primers. Gene. 1982;19:259-68.

14. Durfee T, Nelson R, Baldwin S, Plunkett G, 3rd, Burland V, Mau B, et al. The complete genome sequence of *Escherichia coli* DH10B: insights into the biology of a laboratory workhorse. J Bacteriol. 2008;190(7):2597-606. Epub 2008/02/05. doi: JB.01695-07 [pii]
10.1128/JB.01695-07 [doi]. PubMed PMID: 18245285.

15. Datsenko KA, Wanner BL. One-step inactivation of chromosomal genes in *Escherichia coli* K-12 using PCR products. Proc Natl Acad Sci U S A. 2000;97(12):6640-5.

16. Herrero M, de Lorenzo V, Timmis KN. Transposon vectors containing non-antibiotic resistance selection markers for cloning and stable chromosomal insertion of foreign genes in gram-negative bacteria. J Bacteriol. 1990;172(11):6557-67. PubMed PMID: 2172216.

17. Charpentier X, Oswald E. Identification of the secretion and translocation domain of the enteropathogenic and enterohemorrhagic *Escherichia coli* effector Cif, using TEM-1 beta-lactamase as a new fluorescence-based reporter. J Bacteriol. 2004;186(16):5486-95. PubMed PMID: 15292151.
18. Blanco-Toribio A, Muyldermans S, Frankel G, Fernández LA. Direct injection of functional single-domain antibodies from *E. coli* into human cells. PLoS ONE. 2010;5(12):e15227. Epub 2010/12/21. doi: 10.1371/journal.pone.0015227. PubMed PMID: 21170340; PubMed Central PMCID: PMC2999559.

**Table A.** ***E. coli*** **K-12 strains and plasmids used in this work**

| Name | Relevant genotype and features | Reference |
|---|---|---|
| **DH10B-T1$^{R}$** | (F- λ-) *mcrA* Δ*mrr-hsdRMS-mcrBC* φ80*lacZDM15* Δ*lacX74 recA1 endA1 araD139* Δ*(ara, leu)7697 galU galK rpsL (Str$^{R}$) nupG tonA* | [14] |
| **BW25141** | (F- λ-) Δ*(araD-araB)567*, Δ*lacZ4787*(::rrnB-3), Δ*(phoB-phoR)580*, *galU95*, Δ*uidA3*::*pir*, *recA1*, *endA9*(del-ins)*::FRT*, *rph-1*, Δ*(rhaD-rhaB)568*, *hsdR51* | [15] |
| **CC118 λ*pir*** | Δ*(ara-leu) araD* Δ*lacX74 galE galK phoA20 thi- rpsE rpoB argE(Am) recA1*, λ*pir* | [16] |
| **pCX340** | (Tc$^{R}$) pBR ori, p*trc* promoter, for fusions to the β-lactamase | [17] |
| **pEspF$_{1\text{-}20}$Bla** | pCX340 derivative; EspF residues 1-20 fused to β-lactamase | [18] |
| **pACBSR** | (Cm$^{R}$) p15A ori, $P_{BAD}$ promoter, I-*Sce*I endonuclease and λ Red genes | [2] |
| **pACBSR-Sp** | (Sp$^{R}$) p15A ori, $P_{BAD}$ promoter, I-*Sce*I endonuclease and λ Red genes | [3] |
| **pGE** | (Km$^{R}$) R6K-ori, polylinker flanked with two I-*Sce*I restriction sites | [12] |
| **pGETS** | (Km$^{R}$) pSC101-ts ori, polylinker flanked with two I-*Sce*I restriction sites | [3] |
| **pGEΔ*escN*** | pGE derivative; with HRs flanking *escN* of EPEC | This work |
| **pGETSΔ*map*** | pGETS derivative; with HRs flanking the *map* gene of EPEC | This work |
| **pGEΔ*espG*** | pGE derivative; with HRs flanking the *espG* gene of EPEC | This work |
| **pGETSΔ*espF*** | pGETS derivative; with HRs flanking *espF* gene of EPEC | This work |
| **pGEΔ*espH*** | pGE derivative; with HRs flanking the *espH* gene of EPEC | This work |
| **pGEΔIE5** | pGE derivative; with HRs flanking IE5 cluster *espC* and *espG* of EPEC | This work |
| **pGEΔIE6** | pGE derivative; with HRs flanking IE6 cluster *espL*, *nleB*, *nleE*, *efa1*/*lifA* of EPEC | This work |
| **pGEΔIE2** | pGE derivative; with HRs flanking IE2 cluster *efa1*/*lifA-like, nleE, nleB**, *espL** of EPEC | This work |
| **pGEΔPP2** | pGE derivative; with HRs flanking PP2 cluster *nleH, cif**, *espJ* of EPEC | This work |
| **pGEΔPP3** | pGE derivative; with HRs flanking PP3 gene *nleJ* of EPEC | This work |
| **pGEΔPP4** | pGE derivative; with HRs flanking PP4 cluster *nleG, nleB, nleC, nleH**, *nleD* of EPEC | This work |
| **pGEΔPP6** | pGE derivative; with HRs flanking PP6 cluster *nleA*/*espL, nleH ,nleF, espO* of EPEC | This work |
| **pGEΔPP2-Amp-FRT** | pGE derivative; Amp-FRT and HRs of PP2 cluster *nleH, Cif**, *espJ* of EPEC | This work |
| **pGEΔPP3-Amp-FRT** | pGE derivative; Amp-FRT and HRs of PP3 gene *nleJ* of EPEC | This work |
| **pGEΔPP4-Amp-FRT** | pGE derivative; Amp-FRT and HRs of PP4 cluster *nleG, nleB, nleC, nleH**, *nleD* of EPEC | This work |
| **pGETSΔPP6-Amp-FRT** | pGETS derivative; Amp-FRT and HRs of PP6 cluster *nleA*/*espL, nleH, nleF, espO* of EPEC | This work |
| **pGEΔ*espZ-2*** | pGE derivative; HRs flanking *espZ* gene and its RBS | This work |
| **pGEΔ*tir*** | pGE derivative; HRs flanking *tir* gene of EPEC | This work |
| **pGE+*map*** | pGE derivative; with *map* plus HRs as in the chromosome of EPEC | This work |
| **pGE+*espH*** | pGE derivative; with *espH* plus HRs as in the chromosome of EPEC | This work |
| **pGE+*espF*** | pGE derivative; with *espF* plus HRs as in the chromosome of EPEC | This work |
| **pGE+*espG*** | pGE derivativee; with *espG* plus HRs as in the chromosome of EPEC | This work |
| **pGEΔPP4+*nleC*** | pGEΔPP4 derivative; with *nleC* and intergenic region between *nleG and nleB2* | This work |
| **pGEΔ*eae*** | pGE derivative; with HRs flanking *eae* gene of EPEC | This work |
| **pGEΔ*nleE2*** | pGE derivative; with HRs flanking *nleE2* gene in IE2 of EPEC | This work |
| **pGEΔ*lifA*-like** | pGE derivative; with HRs flanking *efa1*/*lifA-like* gene in IE2 of EPEC | This work |
| **pGEΔ*lifA*** | pGE derivative; with HRs flanking *efa1*/*lifA* gene in IE6 of EPEC | This work |

**Table B. Modifications of EPEC genome using pGE and pGETS plasmids**

| Resulting strain | Parental strain | Plasmid integrated | Checking primers |
|---|---|---|---|
| **EPECΔ*escN*** | EPEC | pGEΔ*escN* | 64,65 |
| **EPECΔ*map*** | EPEC | pGEΔ*map* | 1,2 |
| **EPEC11** | EPECΔ*map* | pGEΔ*espG* | 7,8 |
| **EPEC10** | EPEC11 | pGEΔ*espF* | 9,10 |
| **EPEC9** | EPEC10 | pGEΔ*espH* | 11,14 |
| **EPEC8** | EPEC9 | pGEΔIE5 | 19,20 |
| **EPEC7** | EPEC8 | pGEΔIE6 | 25,26 |
| **EPEC6** | EPEC7 | pGEΔIE2 | 31,32 |
| **EPEC5-Amp-FRT** | EPEC6 | PGEΔPP2-Amp-FRT | 37,38 |
| **EPEC5** | EPEC5-Amp-FRT | PGEΔPP2 | 37,38 |
| **EPEC4-Amp-FRT** | EPEC5 | pGEΔPP3-Amp-FRT | 43,44 |
| **EPEC4** | EPEC4-Amp-FRT | pGEΔPP3 | 43,44 |
| **EPEC3-Amp-FRT** | EPEC4 | pGEΔPP4-Amp-FRT | 49,50 |
| **EPEC3** | EPEC3-Amp-FRT | pGEΔPP4 | 49,50 |
| **EPEC2-Amp-FRT** | EPEC3 | pGETSΔPP6-Amp-FRT | 55,56 |
| **EPEC2** | EPEC2-Amp-FRT | pGEΔPP6 | 55,56 |
| **EPEC1** | EPEC2 | pGEΔ*espZ-2* | 75,76 |
| **EPEC0** | EPEC1 | pGEΔ*tir* | 81,82 |
| **EPEC7Δ*lifA*-like** | EPEC7 | pGEΔ*lifA*-like | 31,89 |
| **EPEC7Δ*nleE2*** | EPEC7 | pGEΔ*nleE* | 94,95 |
| **EPEC2*map*** | EPEC2 | pGE+*map* | 1,2 |
| **EPEC1*map*** | EPEC1 | pGE+*map* | 1,2 |
| **EPEC0*map*** | EPEC0 | pGE+*map* | 1,82 |
| **EPEC2*nleC*** | EPEC2 | pGE+*nleC* | 49,50 |
| **EPEC1*nleC*** | EPEC1 | pGE+*nleC* | 49,50 |
| **EPEC0*nleC*** | EPEC0 | pGE+*nleC* | 49,50 |
| **EPEC2*map+espH*** | EPEC2*map* | pGE+*espH* | 11,14 |
| **EPEC2*map+espH+espF*** | EPEC2*map+espH* | pGE+*espF* | 9,10 |
| **EPEC2*LEE*** | EPEC2*map+espH+espF* | pGE+*espG* | 7,8 |
| **EPECΔ*espZ*-2** | EPEC | EPECΔ*espZ*-2 | 75,76 |
| **EPEC2Δ*eae*** | EPEC2 | pGEΔ*eae* | 106,107 |
| **EPEC1Δ*eae*** | EPEC1 | pGEΔ*eae* | 106,107 |
| **EPECΔ*lifA*-like** | EPEC | pGEΔ*lifA-like* | 31, 89 |
| **EPECΔ*lifA*** | EPEC | pGEΔ*lifA* | 116, 26 |
| **EPECΔ*lifA-like* Δ*lifA*** | EPECΔ*lifA-lke* | pGEΔ*lifA* | 116,26 |

**Table C. Oligonucleotides used in this work (1/4)**

| Number | Name | Sequence (5´-3´) |
|---|---|---|
| **1** | PF.CesF82 | cgtgaaaagcgagggcgtcagtttg |
| **2** | PR-tir.86 | gcgccgtctgtttgtgaaggtagtg |
| **3** | F.XhoI.espG.HR1(2) | ccggttctcgaggcctctggaatagttgcttgctttacactcag |
| **4** | R.espG.HR1.Fus | tatgatgctataataaaactttattaatcaaaaccaataatagaaatc |
| **5** | F.espG.HR2.Fus | taaagttttattatagcatcatatagtgtcaataatatacaagatatttatagcgg |
| **6** | R.sphI.espG.HR2(2) | cgacatgcatgcgaacaagggacaaatagctgaacaagtaaccgcg |
| **7** | F.check.ΔespG | cgattcatcggacagaatcatcagactttcat |
| **8** | R.check.ΔespG | tcggcgaaagaggatctgccatacatcaag |
| **9** | PF.escF.179 | cgataaaagatctggtctcaaccatttctaacc |
| **10** | PR.IE.espF | gtaatacggaaatacattgag |
| **11** | F.XhoI.espH.HR1(3) | cggctcgagccggaaggtgatgtgtcagttgatgatg |
| **12** | PR.EspH.HR1.Fus | cataaaataatactcctgattaatcacatacta |
| **13** | PF.EspH.HR2.Fus | tgattaatcaggagtattattttatgctgttttctttttctcc |
| **14** | R.sphI.espH.HR2 | gcccgcatgcctgacgccctcgcttttcacgataacg |
| **15** | F.XhoI.*espG2*.HR1 | cccgctcgagtgaaggcgaaaaagatgattg |
| **16** | R.SacI.*espG2*.HR1.Fus | cagatttaaacgtctggagctctacacatcctttttattc |
| **17** | F.SacI.*espC*.HR2.Fus | gaataaaaaggatgtgtagagctccagacgtttaaatctggc |
| **18** | R.speI.*espC*.HR2 | ccggactagtcagtagctgaagtaatggtattgc |
| **19** | F.check.ΔIE5 | cttaaccagataagagttaaatg |
| **20** | R.check.ΔIE5 | gccacgggtaaaaagtggcgttttcg |
| **21** | F.XhoI.*espL*.HR1 | ccggctcgaggtgaagccaccctcctctcccctggcg |
| **22** | R.SacI.*espL*.HR1 | cagagattttaagagctcaacaacatttgtgcctgaggaaag |
| **23** | F.SacI.*lifA*.HR2 | cacaaatgttgttgagctcttaaaatctctgttaaagatg |
| **24** | R.SpeI.*lifA*.HR2 | cctagactagtgtcagattctgaccagacg |
| **25** | F.check.ΔIE6 | gtcggtttttccgtcccaccgggatatc |
| **26** | R.check.ΔIE6 | gttaccaattcttgtctaatggc |
| **27** | F.XhoI.*lifA*-like.HR1 | ccggctcgagggtgtgcaggatacctgcctctatcatc |
| **28** | R.SacI.*lifA*-like.HR1 | cataccatctttatggagctctagttttgcacaatatattc |
| **29** | F.SacI.*espL**.HR2 | gtgcaaaactagagctccataaagatggtatgacc |
| **30** | R.SpeI.*espL**.HR2 | cctagactagttcatgattgattagctaaccagg |
| **31** | F.check.ΔIE2 | cagccagaaaaatgtggtgtttaac |
| **32** | R.check.ΔIE2 | ctgtttacctctttctcagggagtttag |
| **33** | F.XhoI.PP2.HR1.n | cccctcgagagtgtgtggtaagaccgctg |
| **34** | R.PP2.HR1.n | gaacgtgaaacgctgagctcccccattatccgagctag |

**Table C. Oligonucleotides used in this work *(continued 2/4)***

| **Number** | **Name** | **Sequence (5´-3´)** |
|---|---|---|
| **35** | F.PP2.HR2.n | cggataatgggggagctcagcgtttcacgttcagc |
| **36** | R.sphI.PP2.HR2 | cgcggcatgcccgcctcctttcctccccggtctg |
| **37** | F.check.ΔPP2 | ctgaagtccggctggagtgagtg |
| **38** | R.check.ΔPP2.n | gggtaatcacgcaggtggtgatac |
| **39** | F.XhoI.nleJ.HR1 | cccgctcgaggttgtaagtaccccgcttaggtg |
| **40** | R.SacI.nleJ.HR1.n | ctactcctcatagagctcttacgggtaaaagcattctttattc |
| **41** | F.SacI.nleJ.HR2.n | gcttttacccgtaagagctctatgaggagtagcaaagtgcactc |
| **42** | R.SphI.nleJ.HR2 | cgcggcatgccatgcaccacctttatccag |
| **43** | F.check.ΔnleJ | gagacataaattcctaactgtg |
| **44** | R.check.ΔnleJ | ggtgaggtacaaccgcaaacac |
| **45** | F.XhoI.PP4.HR1.n | gcgctcgagccgctgcaaatcctgcgtgc |
| **46** | R.SacI.PP4.HR1.n | caagaaacacaggagctcatatgtgatactaaccg |
| **47** | F.SacI.PP4.HR2.n | gtatcacatatgagctcctgtgtttcttgtgc |
| **48** | R.SphI.PP4.HR2.n | cgcggcatgcccggcagacttgctacctgc |
| **49** | F.check.ΔPP4.n | cattctggagtcagatgagaatgg |
| **50** | R.check.ΔPP4 | cagatatcgccctggttgatag |
| **51** | F.XhoI.PP6.HR1 | cccgctcgagatttgtggatgacatttgttgtggacc |
| **52** | R.SacI.PP6.HR1.n | cacactagatcgagctcgttggacaacggcatccaaatc |
| **53** | R.SacI.PP6.HR2.n | ccgttgtccaacgagctcgatctagtgtgattacaatc |
| **54** | R.SphI.PP6.HR2.n | cgcgcatgcccactttaactgcatgacagg |
| **55** | F.check.ΔPP6 | gccgatacagtgcgtggtgaggc |
| **56** | R.check.ΔPP6.n | ggtttacattgttctaccacaatag |
| **57** | F.SacI.HindIII.Amp-FRT | cgcgagctcaagctt<u>gaagttcctatactttctagagaataggaacttcggaataggaacttc</u>atgagtaaacttggtctgac |
| **58** | F.SacI.NdeI.Amp-FRT | cgcgagctccatatg<u>gaagttcctatactttctagagaataggaacttcggaataggaacttc</u>atgagtaaacttggtctgac |
| **59** | R.SacI.SpeI.Amp-FRT | cgcgagctcactagt<u>gaagttcctattccgaagttcctattctctagaaagtataggaacttc</u>tcggggaaatgtgcgcgg |
| **60** | F.XhoI.*escN*.HR1 | ggccctcgagtgtgaaagagctgcagcgccagc |
| **61** | R.SacI.*escN*.HR1 | gggcgagctcttaccgttcctaatactttaag |
| **62** | F.SacI.*escN*.HR2 | gggcgagctcgtatgttggacagaattttatctattcg |
| **63** | R.SphI.*escN*.HR2 | gacatgcatgcgattccgttactacatttaattg |
| **64** | F.check.Δ*escN* | cggcgtacaagaaacgcgttatttg |
| **65** | R.check.Δ*escN* | cgaaaacatagtcttttttatg |
| **66** | F.XhoI.*map*.HR1 | gcgctcgagagatctttgcaaaattgttcattc |
| **67** | R.SphI.*map*.HR2 | gccgcatgcacacgttcctttatattactatg |
| **68** | R.SpeI.espH.HR2 | gcgactagtctgacgccctcgcttttcacgataacg |

**Table C. Oligonucleotides used in this work *(continued 3/4)***

| Number | Name | Sequence (5´-3´) |
|---|---|---|
| **69** | F.XhoI.*espF*.HR1. | ggccctcgagtaagcttcccaaatatgcgg |
| **70** | R.SphI.*espF*.HR2 | cgccgcatgccttcaggaaaaatataaccagataac |
| **71** | F.XhoI.*espZ*.HR1 | ccggttctcgagacagtgcttgcatctgattagcttctttttctg |
| **72** | R.SacI.*espZ*.HR1.new.E | gcgcgagctccataaaaatagagaggtaatggatgcattatgc |
| **73** | F.SacI.*espZ*.HR2.new.E | cgcgcgagctcctaatttagacatttacctgg |
| **74** | R.SphI.*espZ*.HR2 | cgacatgcatgcgcgacaggcgcatcaacgtcgtaatcaac |
| **75** | F.check.Δ*espZ* | cctctttttccacactgagtgtcatatttcc |
| **76** | R.check.Δ*espZ* | gttaccgaaggagtaaataatgtcacccgc |
| **77** | F.XhoI.*tir*.HR1.new | cgcgctcgagggggaaacttactgcgctgttattttttttc |
| **78** | R.SacI.*tir*.HR1.new | gcgcgagctcacatatatccttttatttagaaatttg |
| **79** | F.SacI.*tir*.HR2.Fus | aaggatatatgtgagctcatatatctgtgagtatttag |
| **80** | R.SphI.*tir*.HR2 | cgcggcatgcgtttgggctccaccacaatgag |
| **81** | F.check.Δtir | cgtgaaaagcgagggcgtcagtttg |
| **82** | R.check.Δtir | caatcctaaaccagcactaagc |
| **83** | F.SacI.intergenic.nlGB | gcgcgagctcaataaatattacccaagc |
| **84** | R.Fus.intergenic.nlGG | ctatatcaaattcattcgtcgtcctgtttatcc |
| **85** | F.Fus.*nleC* | gataaacaggacgacgaatgaatttgatatagtttattttg |
| **86** | R.SacI.*nleC* | gcgcgagctcaaaatgtatgaatagtaac |
| **87** | F.SacI.*lifA*-like.HR2 | ggccgagctcctgcaaaggtttagatattaac |
| **88** | R.SpeI.lifA-like.HR2 | ggccactagtagaagctcagcaacttgtgtaagg |
| **89** | R.check.Δ*lifA*-like | gtgaaatgggagaaatctttagctac |
| **90** | F.XhoI.*nleE*.HR1 | ggccctcgagctgcaaaggtttagatattaac |
| **91** | R.SacI.*nleE*.HR1 | cagttcatggtaagagctcagaagctcagcaacttgtg |
| **92** | F.SacI.HR2.*nleE* | gctgagcttctgagctcttaccatgaactgc |
| **93** | R.SpeI.*nleE*.HR2 | ggccactagtccctgccagtgagaggg |
| **94** | F.check.Δ*nleE* | catattccggatgttctttgatac |
| **95** | R.check.Δ*nleE* | gagcagatgtggatttcagcatg |
| **96** | F.AscI.repA101 | cttggcgcgccgttagtcttgatgcttcactg |
| **97** | R.I-*Sce*I.XhoI.oriR101 | cttggcgcgccgttagtcttgatgcttcactg |
| **98** | F.XhoI.PACBSR | gcgccctcgagtttagcttccttagctcctgaaaatctcg |
| **99** | R.SacI.PACBSR | cgccccgagctcttttttaaggcagttattggtgccc |
| **100** | F.XhoI.Sm/Sp | gcgccgctcgaggaaccttgaccgaacgcagcgg |
| **101** | R.SacI.Sm/Sp | gcgccggagctcttatttgccgactaccttggtg |
| **102** | F.XhoI.*eae*.HR1 | ccggctcgagcgttatctgatgccaatgacg |

**Table C. Oligonucleotides used in this work *(continued 4/4)***

| Number | Name | Sequence (5´-3´) |
|---|---|---|
| **103** | R.SacI.*eae*.HR1 | gatatttattaaatgagctcgtttgggctccaccacaatgag |
| **104** | F.SacI.*eae*.HR2 | gtggagcccaaacgagctcatttaataaatatctaatcattg |
| **105** | R.SpeI.eae.HR2 | ggccactagtagatccttgccattataaatgc |
| **106** | F.check.Δ*eae* | gcggaaaaaattggtgttg |
| **107** | R.check.Δ*eae* | caatgaactggcatcag |
| **108** | qPCR-lifAhomo Rev | acgccgtgataaaatactccg |
| **109** | qPCR-lifAhomo For | cgatacaacgcccttcattg |
| **110** | qPCR tir For | cggaatagtctatcggctcatc |
| **111** | qPCR tir Rev | tactttggataccttgccctg |
| **112** | qPCR nleE2 For | atggttgttgtgtacagaaatgac |
| **113** | qPCR nleE2 Rev | ctgcctttaaatctggtaactcataat |
| **114** | F.XhoI.*lifA*.HR1 | gcgcccggctcgagcaggcgcttcacgatacgataacgcg |
| **115** | R. SacI.lifA.HR1 | ccgcgagctcaattacatttccgcttaaaaaattatttatg |
| **116** | F.check.ΔlifA | ccataaaagtcgggaaacacagcgctg |

**Table D. Genome comparison between EPECwt and EPEC1 strains**

| Gene(s) deleted | Start position[a] | End position | Deletion size (bp) |
|---|---|---|---|
| *map* | 4000250 | 4000862 | 612 |
| *espG* | 4019635 | 4020832 | 1197 |
| *espF* | 3987712 | 3988333 | 621 |
| *espH* | 4001701 | 4002232 | 531 |
| *espG2, espC* | 2964706 | 2970366 | 5660 |
| *espL*, *nleB1*, *nleE1*, *efa1/lifA* | 3281976 | 3300236 | 18260 |
| *espL**, *nleB**, *nleE2*, *efa1/lifA-like* | 1129857 | 1144231 | 14374 |
| *nleH1*, *cif**, *espJ* | 767779 | 771747 | 3968 |
| *nleJ* | 863525 | 864073 | 548 |
| *nleG*, *nleB*, *nleC*, *nleH**, *nleD* | 1084531 | 1088543 | 4012 |
| *nleA/espI*, *nleH2*, *nleF*, *espO** | 1432916 | 1438953 | 6037 |
| *espZ* | 4007774 | 4008100 | 326 |

[a] Nucleotide positions referred to the parental WT strain E2348/69
