# Supplementary figures and images for: Attaching and effacing (A/E) lesion formation by enteropathogenic E. coli on human intestinal mucosa is dependent on non-LEE effectors
Source: PLoS Pathog. 2017 Oct 30;13(10):e1006706. doi: 10.1371/journal.ppat.1006706 (PMC5685641; doi:10.1371/journal.ppat.1006706)

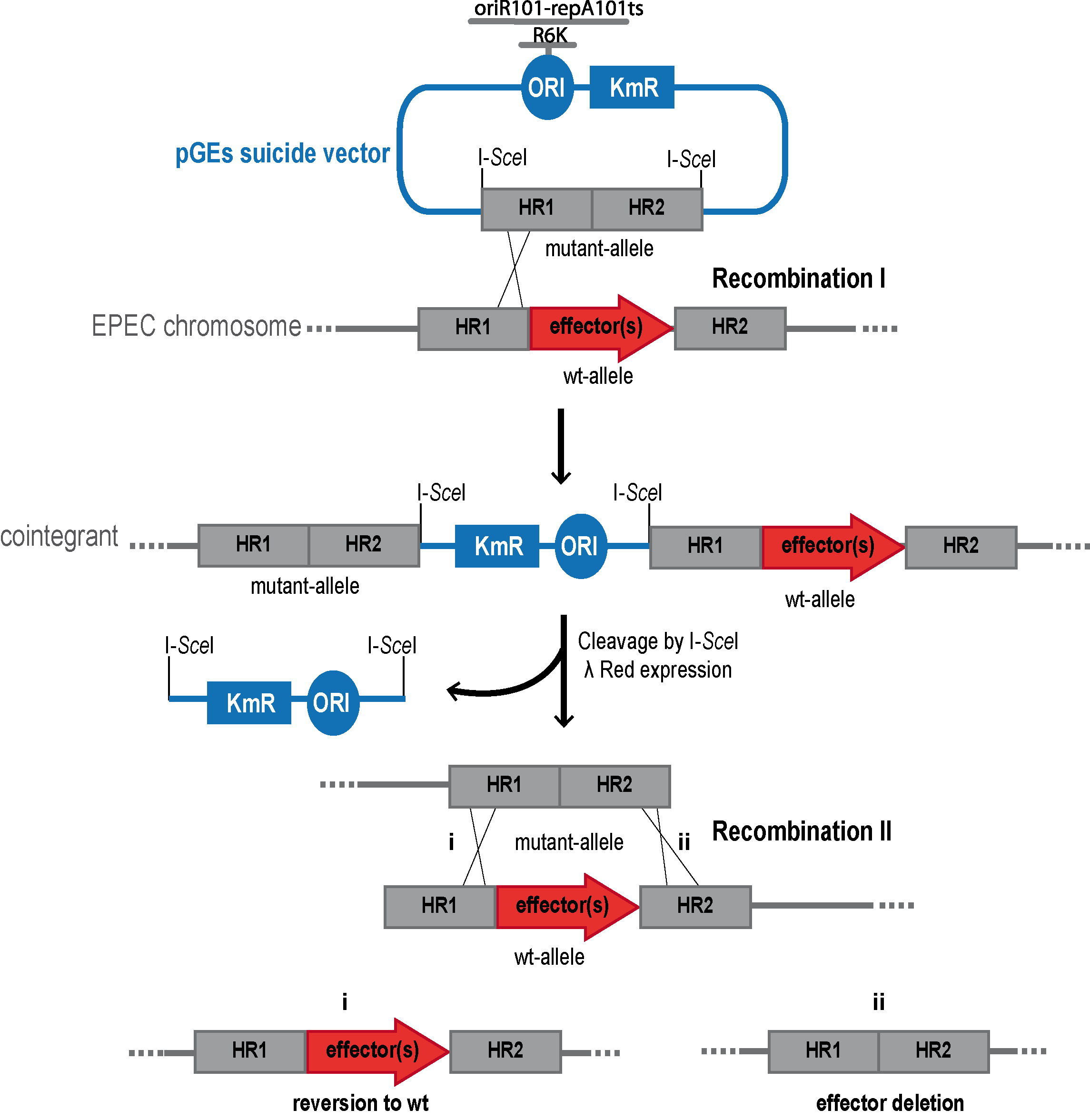

Supplement: S1 Fig — Deletions were carried out by a markerless strategy using suicide plasmids with I-SceI sites and mutant alleles assembled by fusing homology regions (HRs) flanking the targeted effector gene(s). Derivatives of pGE-vector contain an R6K replication origin (π protein dependent) whereas derivatives of pGETS contain a thermo-sensitive replication origin ori101 that replicates at 30°C but not at 37–42°C. The lack of π protein in EPEC, or growth at non-permissive temperature, induce integration of the suicide vector in the chromosome. Co-integrants are identified by the Km resistance phenotype. Expression of the I-SceI in vivo from helper plasmid induces double strand brakes that are repaired by homologous recombination. Depending on the HRs involved in this second recombination, either the WT or the mutant allele can be obtained. Mutants are selected by PCR screening. Mutants do not carry any antibiotic gene marker, vector or recombination sequences ("scars"). (TIF) [file ppat.1006706.s002.tif]

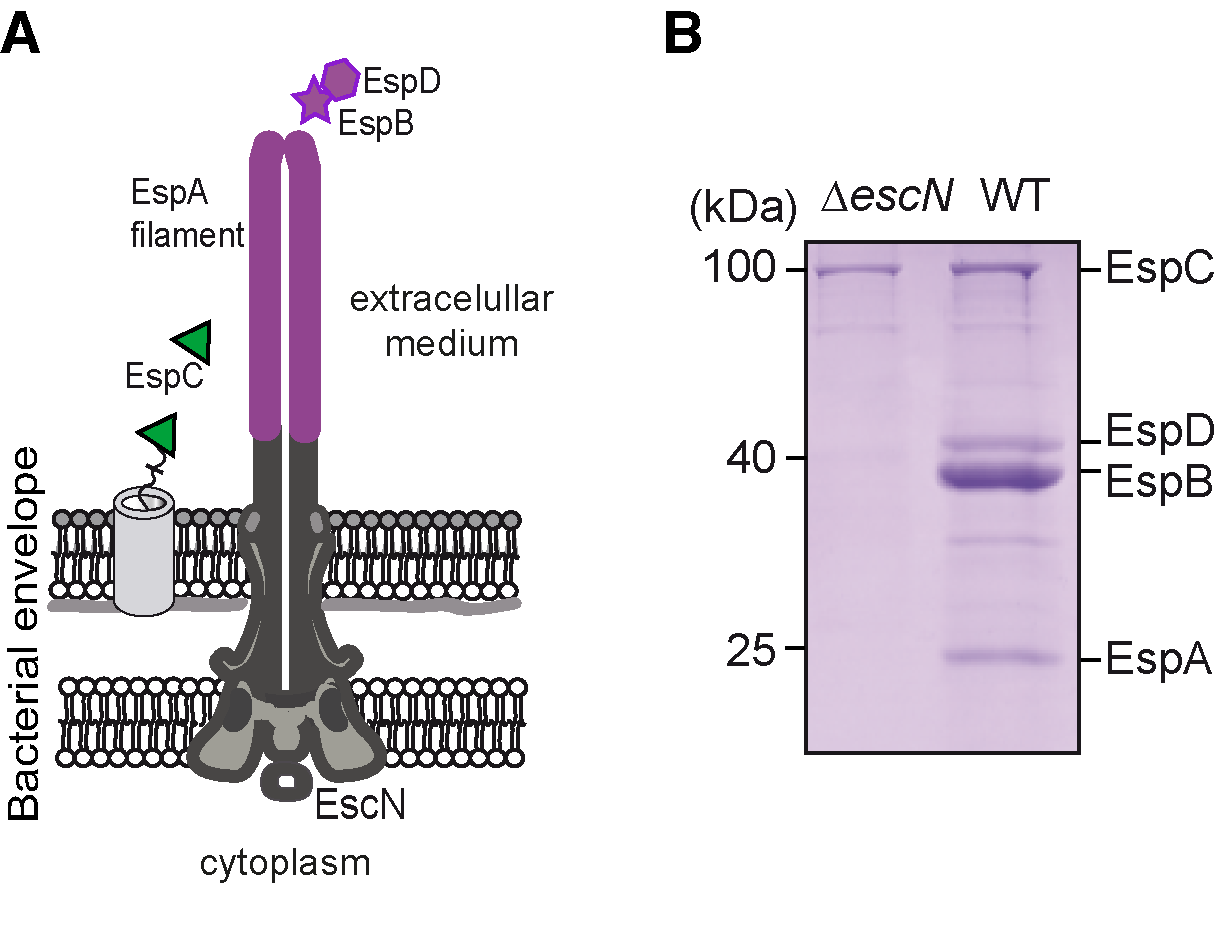

Supplement: S2 Fig — A. Schematic representation of EPEC T3SS injectisome, indicating the filament of EspA, the translocator proteins EspB and EspD, and the ATPase EscN. Secretion of the autotransporter EspC is also shown. B. Coomassie staining of secreted proteins in extracellular media of EPECΔescN and EPEC WT strains grown 4 h at 37°C in DMEM. The translocators EspABD and the autotransporter EspC proteins are labeled. Molecular standards mass proteins are shown in kDa. (TIF) [file ppat.1006706.s003.tif]

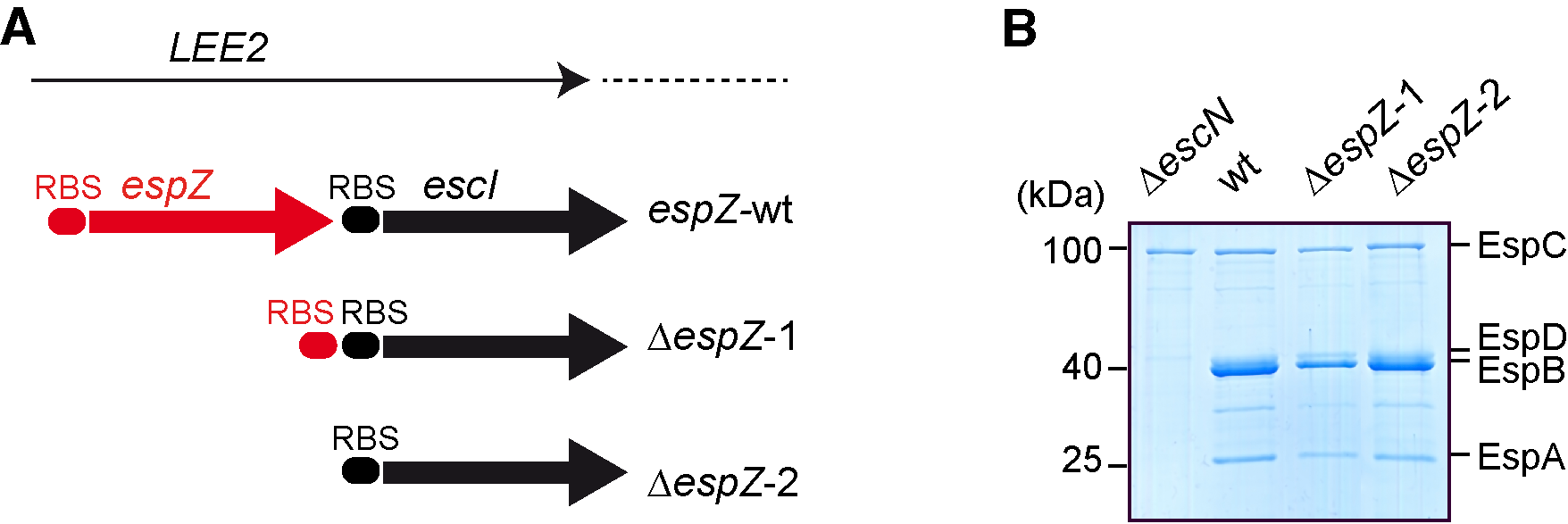

Supplement: S3 Fig — A. Schematic representation of gene organization of espZ and escI, indicating the ribosome binding sites (RBS) in the LEE2 operon of the EPEC WT strain and ΔespZ-1 and ΔespZ-2 mutant alleles. B. Coomassie staining of proteins secreted in the extracellular media of EPECΔescN, EPEC WT, EPECΔespZ-1 and EPECΔespZ-2 strains grown 4 h in DMEM at 37 oC. The translocators EspABD and the autotransporter EspC are labeled. Molecular standards mass proteins are shown in kDa. (TIF) [file ppat.1006706.s004.tif]

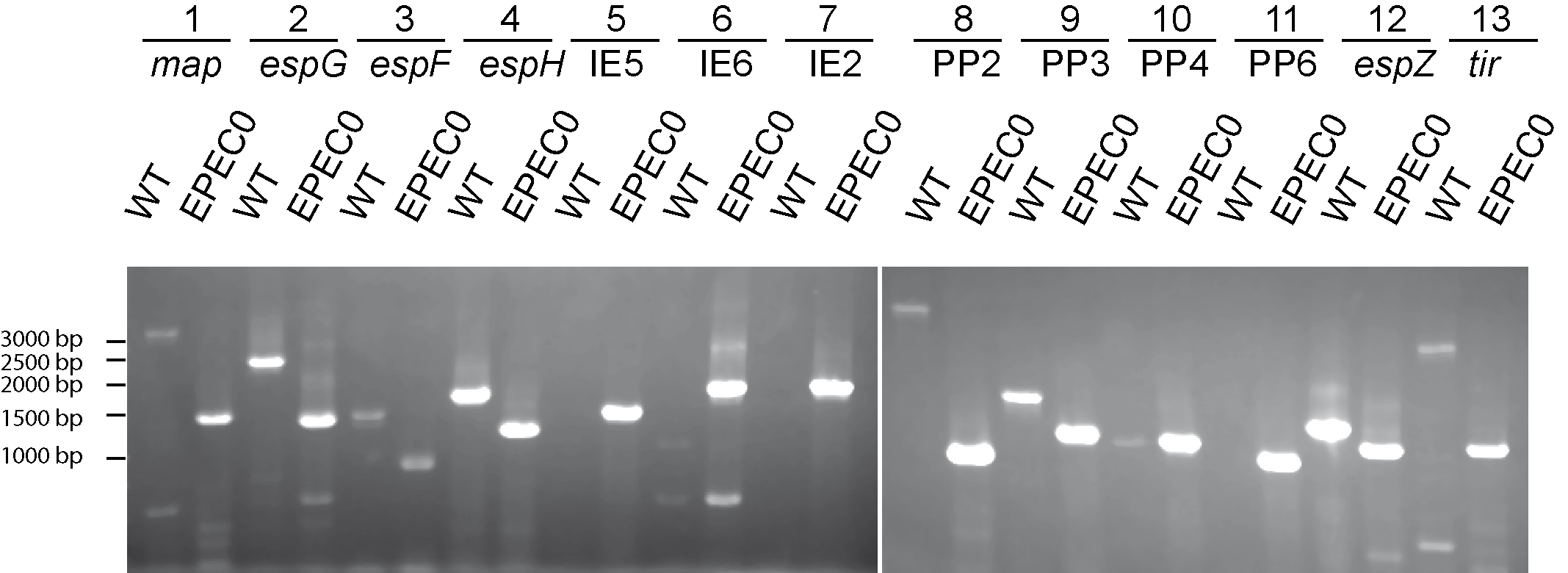

Supplement: S4 Fig — Agarose gel electrophoresis of PCR products amplified from EPEC WT and EPEC0 strains using primers to check deletions described in Tables B and C of S1 Text. The lanes corresponding to a deletion of an effector gene or gene cluster are indicated on top. The order of the deletions is also numbered on top. DNA bands corresponding to amplicons from IE5, IE6, IE2, PP4 and PP6, in the EPECwt strain are not visible given their poor amplification due to their large size. DNA markers are labeled on the left (in bp). (TIF) [file ppat.1006706.s005.tif]

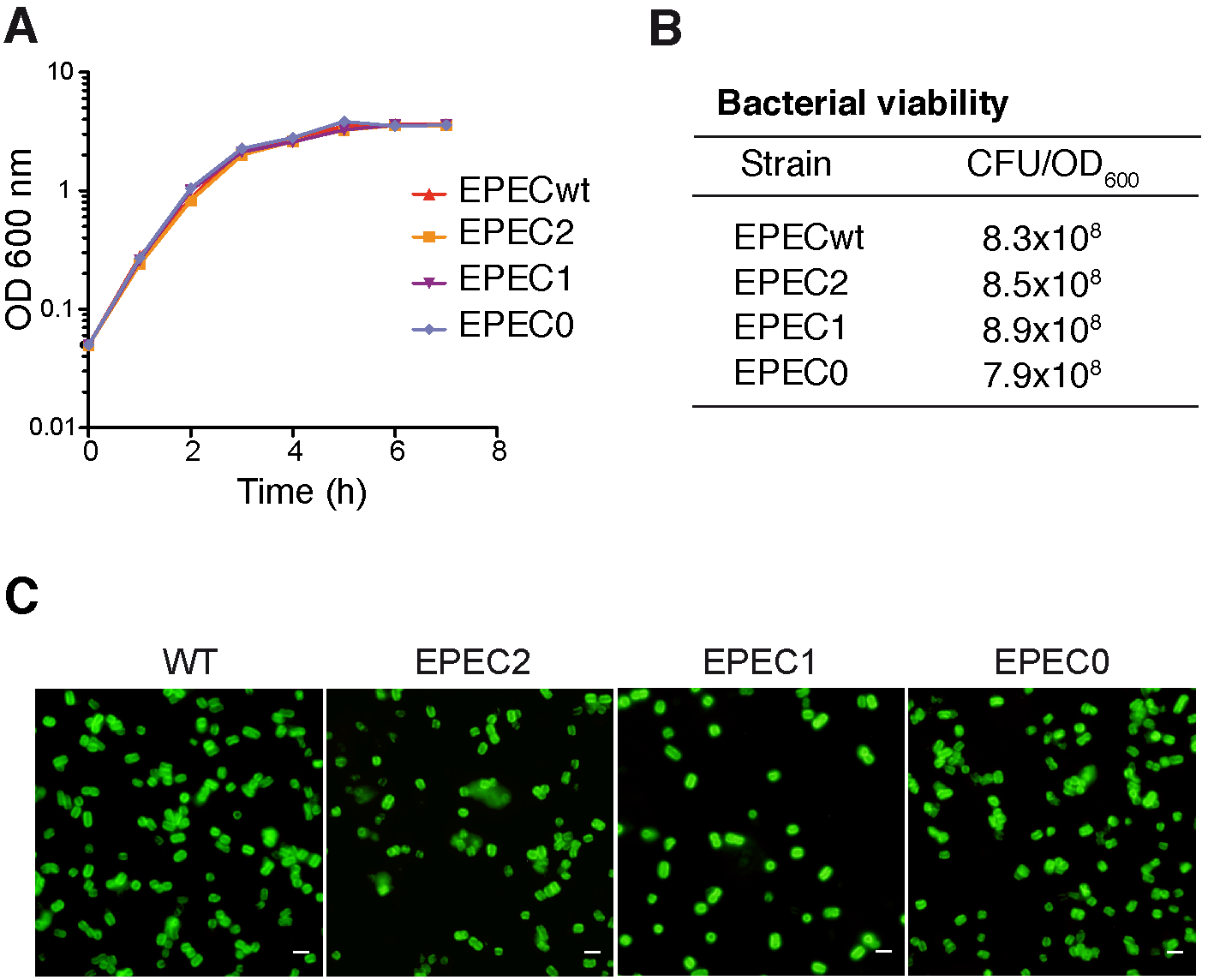

Supplement: S5 Fig — A. Growth curves of EPEC WT, EPEC2, EPEC1 and EPEC0 strains in LB following the optical density (OD) at 600nm at the indicated times points. B. Bacterial viability (CFU/OD600) of the indicated strains (EPEC WT, EPEC2, EPEC1 and EPEC0) grown in DMEM 4 h and plated in LB-agar. C. Immunofluorescence microscopy of bacteroa from EPEC WT, EPEC2, EPEC1 and EPEC0 strains stained with anti-intimin-280 polyclonal serum (green). Scale bar 2 μm. (TIF) [file ppat.1006706.s006.tif]

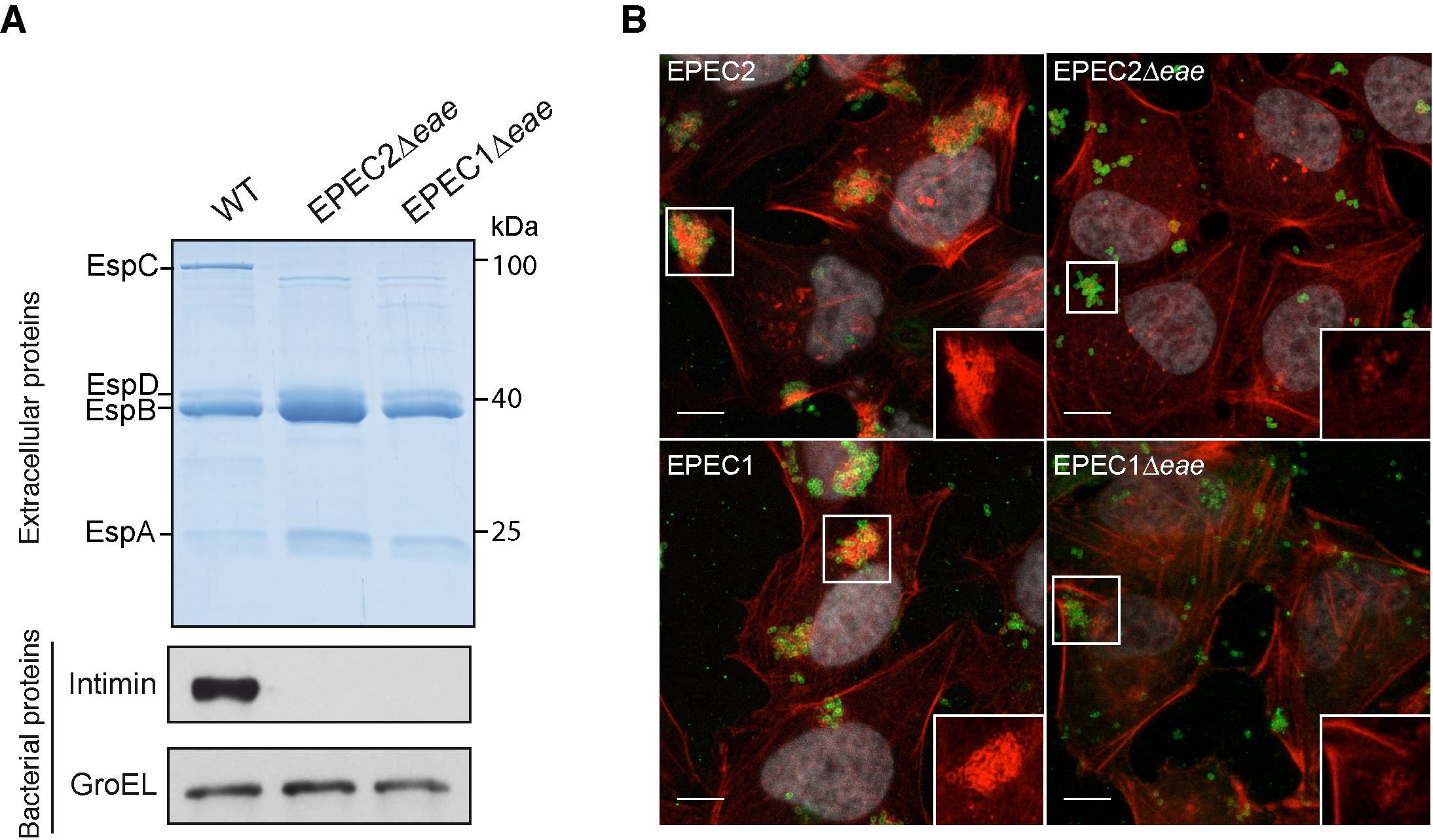

Supplement: S6 Fig — A. Top panel: Coomassie staining of proteins secreted in the extracellular media by EPEC WT, EPEC2Δeae and EPEC1Δeae grown 4 h in DMEM at 37°C. The translocators EspABD and the autotransporter EspC are labeled. Protein standards are labelled in kilodaltons (kDa). Bottom panels: Western blots of bacterial lysates detected with rabbit polyclonal anti-intimin-280 and GroEL (as loading control). B. Immunofluorescence confocal microscopy of HeLa cells infected 90 min with EPEC2, EPEC2Δeae, EPEC1 and EPEC1Δeae. EPEC bacteria are labeled with anti-E. coli (green). Actin is labeled with TRITC phalloidin (red) and cell nuclei are labeled with DAPI (gray). Scale bar 5 μm. (TIF) [file ppat.1006706.s007.tif]

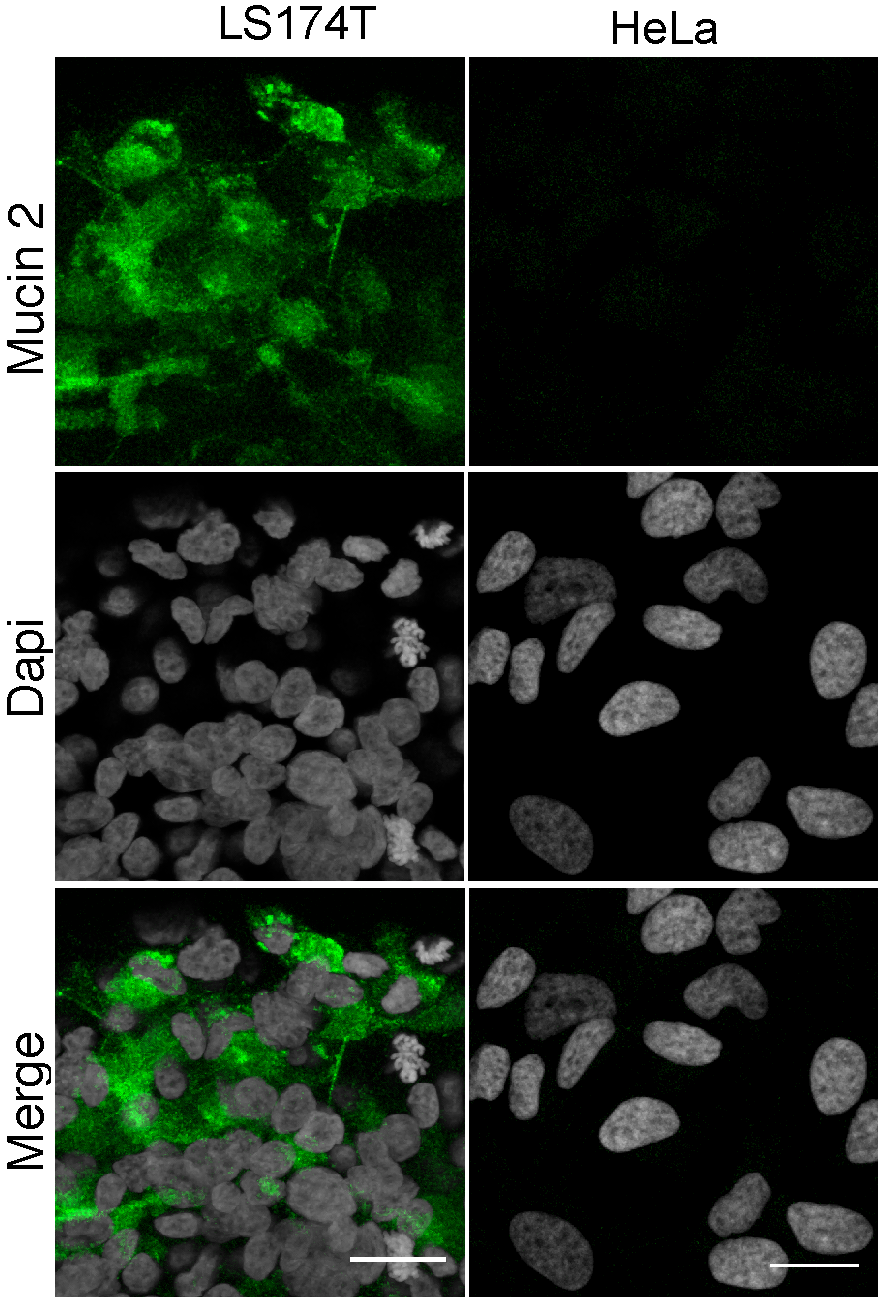

Supplement: S7 Fig — Immunofluorescence microscopy of LS174T and HeLa cells stained with anti-MUC2 rabbit-polyclonal antibody (green) and cell nuclei labeled with DAPI (gray). Scale bar 20 μm. (TIF) [file ppat.1006706.s008.tif]

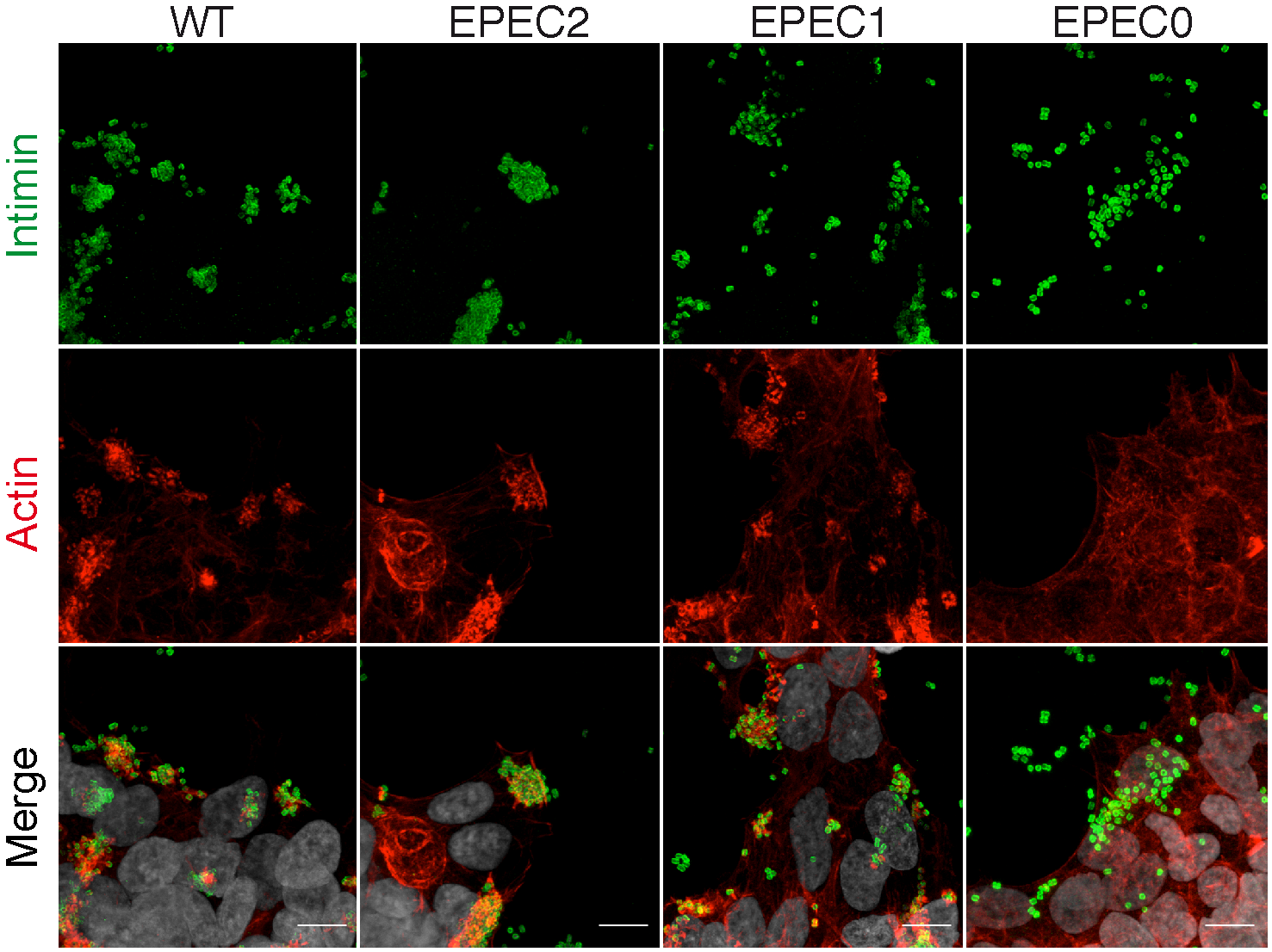

Supplement: S8 Fig — Immunofluorescence confocal microscopy of LS174T cells infected for 90 min with WT EPEC, EPEC2, EPEC1 and EPEC0. EPEC bacteria are labeled with anti-intimin-280 polyclonal serum (green), actin is labeled with TRITC-phalloidin (red) and cell nuclei are labeled with DAPI (gray). Actin polymerization beneath the adherent bacteria is observed in WT EPEC, EPEC2 and EPEC1 strains. Scale bar 10 μm. (TIF) [file ppat.1006706.s009.tif]

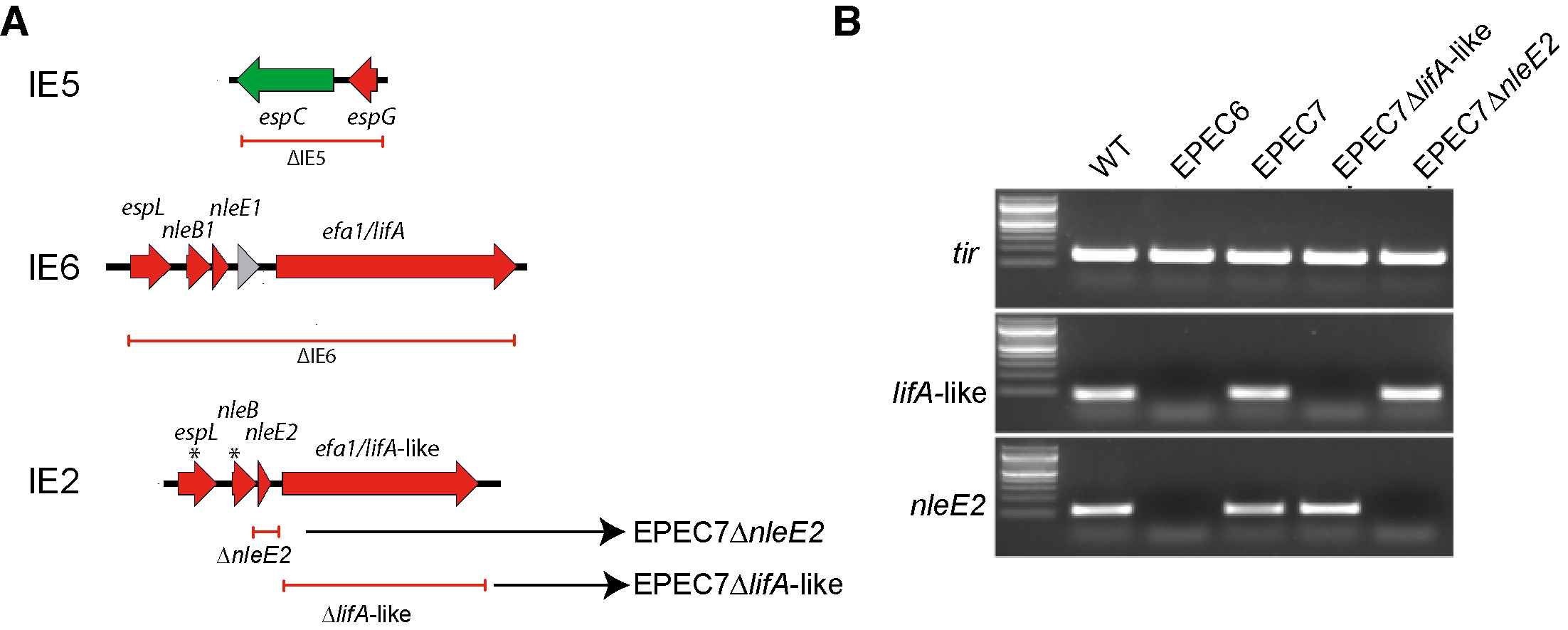

Supplement: S9 Fig — A. Clusters of effectors in IE5, IE6 and IE2. Red bars indicated deletion of IE5 and IE6 encoded-effectors and individual deletion of nleE2 or lifA-like in EPEC7ΔnleE2 and EPEC7ΔlifA-like, respectively. B. Agarose gel electropheresis of RT-PCR products of expression of lifA-like and nleE2 in effectors mutant strains. EPEC7ΔlifA-like strain has normal expression of nleE2. EPEC2ΔnleE2 has normal expression of lifA-like. EPEC7 has expression of lifA-like and nleE2. EPEC6 does not have expression of lifA-like and nleE2. The expression of tir was used as a control for RT-PCR. (TIF) [file ppat.1006706.s010.tif]

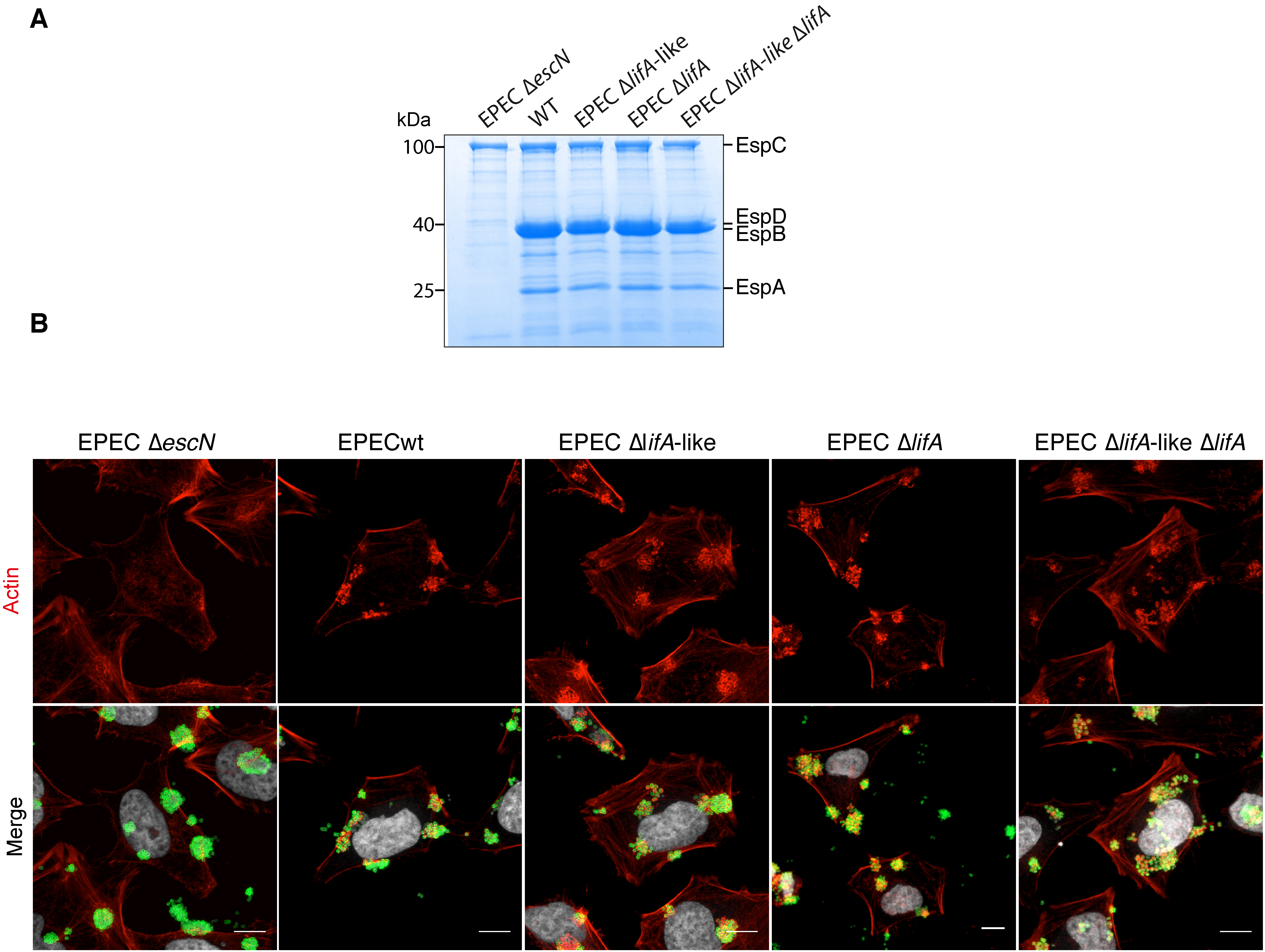

Supplement: S10 Fig — A. Coomassie staining of proteins secreted in the extracellular medium in the indicated EPEC strains grown in DMEM at 37 oC. Protein bands corresponding to the translocators EspA, EspB, EspD and the autotransporter EspC are labelled. Molecular standards are shown in kDa B. Immunofluorescence confocal microscopy of HeLa cells infected for 90 min with WT EPEC, EPECΔescN, EPECΔlifA-like, EPECΔlifA and EPECΔlifA-likeΔlifA strains. EPEC bacteria are labeled with anti-intimin-280 polyclonal serum (green), actin is labeled with TRITC-phalloidin (red) and cell nuclei are labeled with DAPI (gray). Actin polymerization beneath adherent bacteria is observed in WT EPEC, EPECΔlifA-like, EPECΔlifA and EPECΔlifA-likeΔlifA. Scale bar 10 μm. (TIF) [file ppat.1006706.s011.tif]

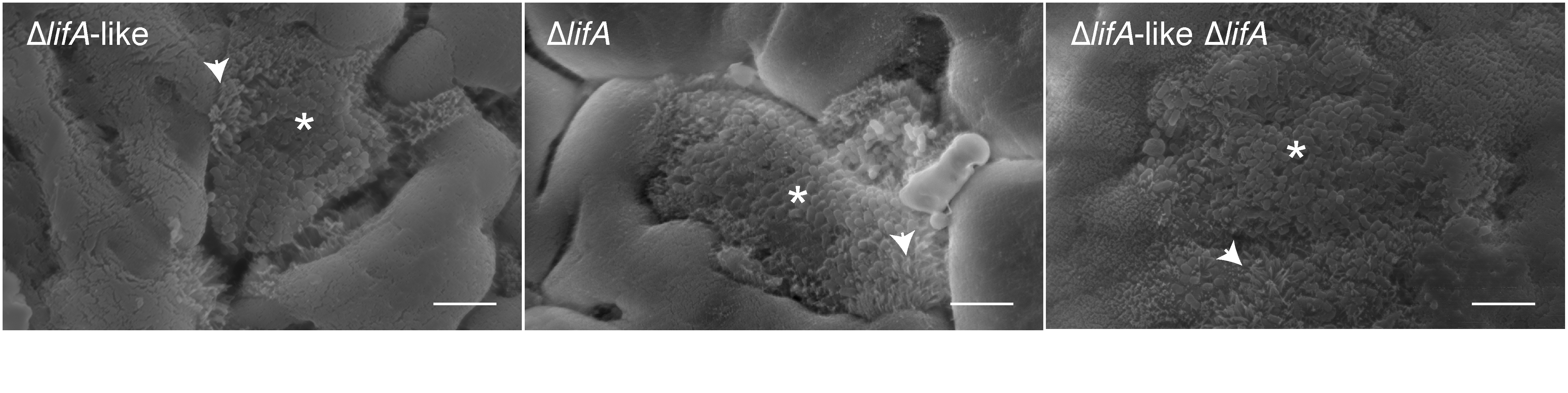

Supplement: S11 Fig — Scanning electron micrographs of human duodenal biopsies infected with EPECΔlifA-like, EPECΔlifA, and double mutant EPECΔlifA-likeΔlifA, showing A/E lesions formed in the intestinal mucosal surface. Large bacterial microcolonies (asterisk) and elongation of microvilli at the periphery of the microcolony (arrowheads) are labeled. Scale bar 5 μm. (TIF) [file ppat.1006706.s012.tif]
